# Fabrication of 3D Biofunctional Magnetic Scaffolds by Combining Fused Deposition Modelling and Inkjet Printing of Superparamagnetic Iron Oxide Nanoparticles

**DOI:** 10.1007/s13770-025-00711-2

**Published:** 2025-03-18

**Authors:** Manuel Estévez, Elisa Batoni, Mónica Cicuéndez, Amedeo Franco Bonatti, Tamara Fernández-Marcelo, Carmelo De Maria, Blanca González, Isabel Izquierdo-Barba, Giovanni Vozzi

**Affiliations:** 1 Departamento de Química en Ciencias Farmacéuticas, Facultad de Farmacia, Instituto de Investigación Sanitaria, Universidad Complutense de Madrid, Hospital 12 de Octubre i+12, Plaza Ramón y Cajal s/n, 28040 Madrid, Spain; 2 Department of Information Engineering, University of Pisa, Via Girolamo Caruso 16, 56122 Pisa, Italy; 3 Research Center “E. Piaggio”, University of Pisa, Via Largo Lucio Lazzarino 1, 56122 Pisa, Italy; 4 Departamento de Química en Ciencias Farmacéuticas, Facultad de Farmacia, Instituto de Investigación Sanitaria del Hospital Clínico San Carlos (IdISSC), Universidad Complutense de Madrid, 28040 Madrid, Spain; 5 Departamento de Bioquímica y Biología Molecular, Facultad de Farmacia, Universidad Complutense de Madrid, Plaza Ramón y Cajal, s/n, 28040 Madrid, Spain; 6 Centro de Investigación Biomédica en Red de Bioingeniería, Biomateriales y Nanomedicina (CIBER-BBN), 28029 Madrid, Spain

**Keywords:** Bone tissue engineering, Inkjet printing, Fused deposition modelling, Superparamagnetic iron oxide nanoparticles, Mechanotransduction

## Abstract

**Background:**

Recently, magnetic composite biomaterials have raised attention in bone tissue engineering as the application of dynamic magnetic fields proved to modulate the proliferation and differentiation of several cell types.

**Methods:**

This study presents a novel method to fabricate biofunctional magnetic scaffolds by the deposition of superparamagnetic iron oxide nanoparticles (SPIONs) through thermal Drop-On-Demand inkjet printing on three-dimensional (3D) printed scaffolds. Firstly, 3D scaffolds based on thermoplastic polymeric composed by poly-L-lactic acid/poly-caprolactone/poly(3-hydroxybutyrate-co-3-hydroxyvalerate) were fabricated by Fused Deposition Modelling. Then, in a second step, SPIONs were incorporated onto the surface of the scaffolds by inkjet printing following a designed 2D pattern.

**Results:**

A complete characterization of the resulting magnetic scaffolds was carried out attending to the surface SPIONs deposits, demonstrating the accuracy and versatility of the production technique, as well as the stability under physiological conditions and the magnetic properties. Biological evaluation with human bone marrow mesenchymal stems cells demonstrated biocompatibility of the scaffolds and increased osteogenic capability under the application of a magnetic field, due to the activation of mechanotransduction processes.

**Conclusion:**

These results show that the developed 3D magnetic biofunctional scaffolds can be a very promising tool for advanced and personalised bone regeneration treatments.

**Graphical abstract:**

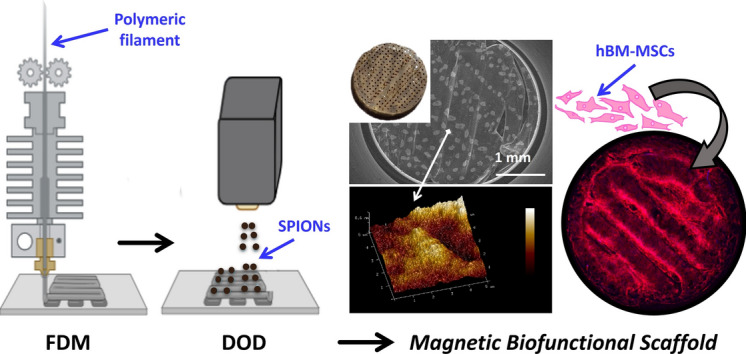

**Supplementary Information:**

The online version contains supplementary material available at 10.1007/s13770-025-00711-2.

## Introduction

Bone disorders and diseases are a worldwide clinical challenge which is expected to rise due to the increasing life expectancy [1, 2]. Traditionally, the most common treatments for bone defects have focused on transplantation (*i.e.*, auto-, allo-, and xenograft); however, cost, infections and the number of compatible donors limit this approach [2]. Bone Tissue Engineering (BTE) represents an alternative to these traditional techniques and is defined as a multidisciplinary field aiming to find new solutions for the healing, repairing and regeneration of bone tissue. The essential components are the scaffold, cell sources, and biochemical and physical stimuli necessary to guide cell differentiation towards the target tissue [2]. A scaffold is defined as a three-dimensional (3D) highly interconnected porous structure which supports cell adhesion, growth, and function providing an environment like the natural Extracellular Matrix (ECM) [3]. Specific cell sources should be appropriately chosen according to the target tissue, and suitable cell culture conditions should be provided to guide their differentiation and maintain their stability and growth [4].

Bone is a complex connective tissue playing different roles in the human body such as mechanical support, organ protection and blood production. Due to the complexity of bone tissue, it is still challenging to develop a bone substitute which resembles the mechanical and biological properties of the native bone ECM, as well as its functionality, to promote the healing and regeneration of the damaged site. In the last decades, great attention has been focused on the development of advanced fabrication technologies and on the synthesis of materials with suitable mechanical strength and stiffness, and with bioactive properties to promote the formation of healthy bone [5]. In BTE, Additive Manufacturing (AM) technologies have been widely used to fabricate bone scaffolds because of their higher accuracy, reproducibility and variability compared to conventional techniques (e.g., freeze-drying, solvent casting) [6, 7]. One of the most used AM technologies in bone scaffold fabrication is Fused Deposition Modelling (FDM), where a thermoplastic polymer is automatically deposited layer-by-layer to create a 3D structure starting from a digital model [8]. In the literature, a great variety of thermoplastic biodegradable polymers have been used to fabricate bone scaffolds through FDM such as polylactic acid (PLA), poly(lactic-co-glycolic) acid (PLGA), poly-caprolactone (PLC), and their blends [9]. Besides, composite formulations comprising different thermoplastic polymers and a ceramic phase (e.g., PLA and calcium-phosphate) have been also developed to enhance the osteogenic potential and the mechanical properties of scaffolds [10].

Recently, magnetic composite biomaterials have raised attention in BTE as the application of dynamic magnetic fields proved to modulate the proliferation and differentiation of several cell types such as Bone Marrow Mesenchymal Stem Cells (BM-MSCs) and mouse-calvaria MC3T3-E1 [11]. Thus, by incorporating low amounts of magnetic material (around 0.07 mg/mL per scaffold), the fabricated scaffolds can be provided with a magnetic response and then remotely manipulated by applying low-intensity magnetic fields (14 ± 2 mT) [12, 13]. In this context, Superparamagnetic Iron Oxide Nanoparticles (SPIONs) have been widely used because of their unique physical and chemical properties and their excellent biocompatibility. Under the action of a magnetic field, SPIONs loaded scaffold can effectively promote osteoblast proliferation, differentiation, as well as angiogenesis [12, 14]. Different strategies have been investigated to synthesize magnetic scaffolds containing SPIONs such as freeze-drying, electrospinning, 3D printing, and chemical synthesis [12, 15]. In the chemical synthesis methods, SPIONs are usually integrated into the scaffolds by infiltration or adsorption. For instance, Samal et al*.* [16] integrated magnetic nanoparticles into silk fibroin scaffolds via the dip-coating method. In another study, silk fibroin scaffolds decorated with SPIONs were obtained by free radical polymerization to accelerate the osteogenic response process through the synergistic effect of magnetic 3D structures in response to weak static magnetic fields [17]. Similarly, magnetic hydroxyapatite (HAp) scaffolds were prepared by immersing HAp scaffolds into a SPIONs solution allowing the infiltration of the magnetic particles into the scaffold’s pores through capillary forces [18]. Thanks to the advantages of 3D printing over conventional scaffold fabrication techniques, researchers have been experimenting with the possibility of directly printing mixtures of biomaterials comprising SPIONs. For instance, Zhang et al*.* [19] 3D printed an injectable paste composed of SPIONs/Mesoporous bioactive glasses/PCL to fabricate 3D magnetic composite scaffolds, which showed multifunctional properties of enhanced osteogenic activity, local anticancer drug delivery and magnetic hyperthermia. Similarly, Das et al*.* [20] prepared a SPION-loaded hydrogel 3D printed into scaffolds to promote osteochondral tissue regeneration via an external magnetic actuator. Another example has been reported by Shokouhimehr et al*.* [21], who 3D printed a hyperplastic bone scaffold incorporated with SPIONs to enhance the bacteriostatic properties of bone grafts for large non-healing bone fractures. Recently, 3D bioprinting of cell-loaded inks (termed bioink [22]) has gained interest also in the development of magnetic scaffolds. A bioink comprising alginate, methylcellulose, and magnetite microparticles was developed to fabricate cell-laden scaffolds that could be deformed by the application of an external magnetic field, thus providing mechanical stimulation to the cells within the construct [23].

In this study, we present a novel method to fabricate magnetic scaffolds by the deposition of SPIONs through thermal Drop-On-Demand (DOD) inkjet printing on 3D printed FDM scaffolds. Inkjet printing has been widely applied in the biomedical field, also for the manipulation of biological materials such as biomaterials, biomolecules and cells [24]. Among them, thermal DOD inkjet printing is considered the most suitable because of its fast-printing speed, high resolution, and low equipment cost [24, 25]. The working principle is based on a heat actuator which heats the ink locally for several microseconds. Thus, the ink is locally vaporized, and heat bubbles are produced and expanded generating a driving force to eject the ink through the nozzles [24, 26, 27]. In the literature, many studies have reported the use of inkjet printing of small molecules, dissolved or suspended in aqueous or organic solution, to be locally deposited on a substrate [24, 26]. Compared to existing coating methods, such as dip-coating, inkjet printing offers the advantage to accurately and precisely depositing specific amounts of material and to control their position following a desired pattern [28–30]. However, to the best of our knowledge SPIONs have never been incorporated through DOD. Herein, we evaluated the possibility of depositing SPIONs in an aqueous solution through thermal DOD inkjet printing to fabricate magnetic scaffolds combining the advantages of both 3D printing of bone scaffolds and inkjet printing of small molecules. Firstly, scaffolds were 3D printed with a thermoplastic polymeric blend filament composed of poly-L-lactic acid (PLLA/PCL/poly[3-hydroxybutyrate-co-3-hydroxyvalerate] (PHBV) (95/5/5 wt%), since its biocompatibility and *in vitro* osteogenic potential reported in previous studies [31, 32]. In a second step, SPIONs loaded solution was inkjet printed on the scaffold surface to provide them with a magnetic functional property. A complete characterization of the resulting magnetic scaffolds was carried out in terms of morphology, surface, magnetic properties, and *in vitro* degradation. Then, *in vitro* biocompatibility and osteogenic effect was performed on human bone marrow mesenchymal stems cells (hBM-MSCs).

## Materials and methods

### Reagents

Sodium oleate 82%, FeCl_3_·6H_2_O 97%, oleic acid 90%, octadecene 90%, dimercaptosuccinic acid 98% (DMSA), dimethyl sulfoxide ≥ 99.9% (DMSO), 12 kDa cellulose membrane, Arg-Gly-Asp (RGD) peptide ≥ 97%, N-(3-dimethylaminopropyl)-N’-ethylcarbodiimide hydrochloride (EDC·HCl) ≥ 98% and *N*-hydroxysulfosuccinimide sodium salt ≥ 98% were purchased from Sigma-Aldrich (Madrid, Spain). Absolute ethanol 99.5%, hexane 95% and toluene 99.5% were purchased from PanReac (Barcelona, Spain). All other chemicals (NaOH, HNO_3_ 65%, HCl 37%, NaCl, MgCl_2_, etc.) were of the highest quality commercially available and used as received. Milli-Q water (resistivity 18.2 MΩ cm at 25 °C) was used in all experiments. 3-polymer blend filaments composed of poly-L-lactic acid (PLLA), polycaprolactone (PCL) and poly(3-hydroxybutyrateco-3-hydroxyvalerate) (PHBV), PLLA/PCL/PHBV (90/5/5 wt%) were kindly provided by University of Newcastle by a previously described protocol [31].

Cells, culture media and biological reagents for *in vitro* cell assays were as follows: human bone marrow derived mesenchymal stem cells (hBM-MSCs) (donor 38,157), mesenchymal stem cell basal medium (MSCBM), mesenchymal cell growth supplement (MCGS), *L*-glutamine, gentamicin sulphate-amphotericin (GA-1000), hMSC osteogenic differentiation medium, trypsin/EDTA 0.25% and phosphate buffered saline (PBS, pH 7.4) were purchased from Lonza and Gibco, Thermo Fisher Scientific, Wilmington, DE, USA. Propidium iodide 1.0 mg/mL solution in water was purchased from Life Technologies Molecular Probes (Thermo Fisher Scientific). 2′,7′-dichlorodihydrofluorescein diacetate (H_2_DCFDA) was purchased from Invitrogen, Thermo Fisher Scientific (Waltham, MA, USA). Alizarin red S, cetylpyridinium chloride, WST-8 cell proliferation kit, paraformaldehyde, triton X-100, Phalloidin-Atto 565 and 4′,6-diamidine-2′-phenylindole dihydrochloride (DAPI) were purchased from Sigma-Aldrich.

### SPIONs and scaffolds characterization

The analytical methods used to characterise the synthesised SPIONs were the following: induction coupled plasma atomic emission spectroscopy (ICP-AES), X-ray diffraction (XRD), transmission electron microscopy (TEM), Fourier transform infrared spectroscopy (FTIR), chemical microanalysis, magnetometry with SQUID-type sensor, electrophoretic mobility measurements to calculate zeta potential (ζ-potential) values and dynamic light scattering (DLS). The physico-chemical characterization of the scaffolds was performed by scanning electron microscopy (SEM), atomic force microscopy (AFM), magnetometry with SQUID-type sensor and ICP-AES to quantify the initial Fe content and to perform a stability test. The equipment and conditions used are described in the Supplementary Material.

### Preparation of SPIONs suspensions

In this work, two aqueous suspensions of SPIONs (SPIONs-DMSA and SPIONs-DMSA-RGD) were prepared. The starting iron oxide nanoparticles were synthesised by the thermal decomposition method following a previously described procedure [33]. First, iron(III) oleate was synthesised from sodium oleate and FeCl_3_·6H_2_O. Then, the nanoparticles were obtained by mixing iron(III) oleate (as iron-containing precursor), oleic acid (as stabiliser) and octadecene (as solvent) and subjecting the mixture to 315 °C. The resulting product was washed with ethanol several times and suspended in toluene. The as-synthesised SPIONs were then transferred to an aqueous medium by ligand exchange, stirring a mixture of DMSA dissolved in DMSO and the suspension of SPIONs in toluene for 3 days. Afterwards, the DMSA-coated nanoparticles were recovered and washed with ethanol. They were then suspended in distilled water, pH adjusted to 10 and dialysed against distilled water. Finally, the pH was adjusted to 7. The colloidal aqueous suspension of DMSA-coated SPIONs obtained was denoted as SPIONs-DMSA. Subsequently, the RGD peptide was covalently attached to the carboxyl groups present on the DMSA coating of the SPIONs by EDC/sulfoNHS chemistry following the procedure described recently [34]. The resulting suspension was named SPIONs-DMSA-RGD. Detailed methodology can be found in the Supplementary Material.

### Printing platform

Scaffolds were 3D fabricated with a novel printing platform characterized by high precision and repeatability in positioning, featured with multiple toolheads for multimaterial and multiscale printing and controlled through LinuxCNC, open-source software for controlling CNC machines (see Fig. S1 in Supplementary Material) [35–37]. Scaffolds were fabricated using two modules: (i) a FDM toolhead, for 3D printing scaffolds with a thermoplastic blend filament, and (ii) a thermal DOD inkjet cartridge, for the precise and local deposition of low viscous ink solutions loaded with SPIONs over the scaffold surface. The FDM module is composed of a 3D printhead (Revo™ Hemera, E3D) equipped with a 0.4 mm brass nozzle and a heated bed, while the inkjet printhead is composed of a cartridge characterized by 12 nozzles and 96 dots per inch (DPI) resolution (model C6602A from HP, HP Extended TIJ 1.0 Print Cartridges), with an ejected ink drop volume of 160 pL [38]. The printhead and the heated bed temperatures are controlled by a PID loop through an Arduino Uno board and two different thermistors. On the other hand, the inkjet nozzles are controlled through an Arduino Nano board to send the electric pulses to fire specific nozzles. To control the two different modules (*i.e.*, FDM and thermal DOD inkjet) together, specific upgrades were made to the printer control software. In detail, three new user *M* commands [36] were created to automatically control the FDM module (*i.e.*, set printhead and bed temperature command) and the inkjet one (*i.e.*, jetting command) through the G-Code.

### Scaffold design and fabrication

#### Design and 3D printing

Cylindrical scaffolds were designed in *Fusion360®* (Autodesk Inc) with a 5 mm diameter and 0.6 mm height to place them inside a 96-multiwell plate for *in vitro* tests. The design was exported as a*.STL* file and sliced in *Slic3r* [39] with 0.2 mm layer height, one external perimeter, and 100% rectangular infill, with 90-degree alternating lines for each layer. An outer skirt around the scaffold was added to clean the nozzle from excess material between one print session and another.

**Fig. 1:**
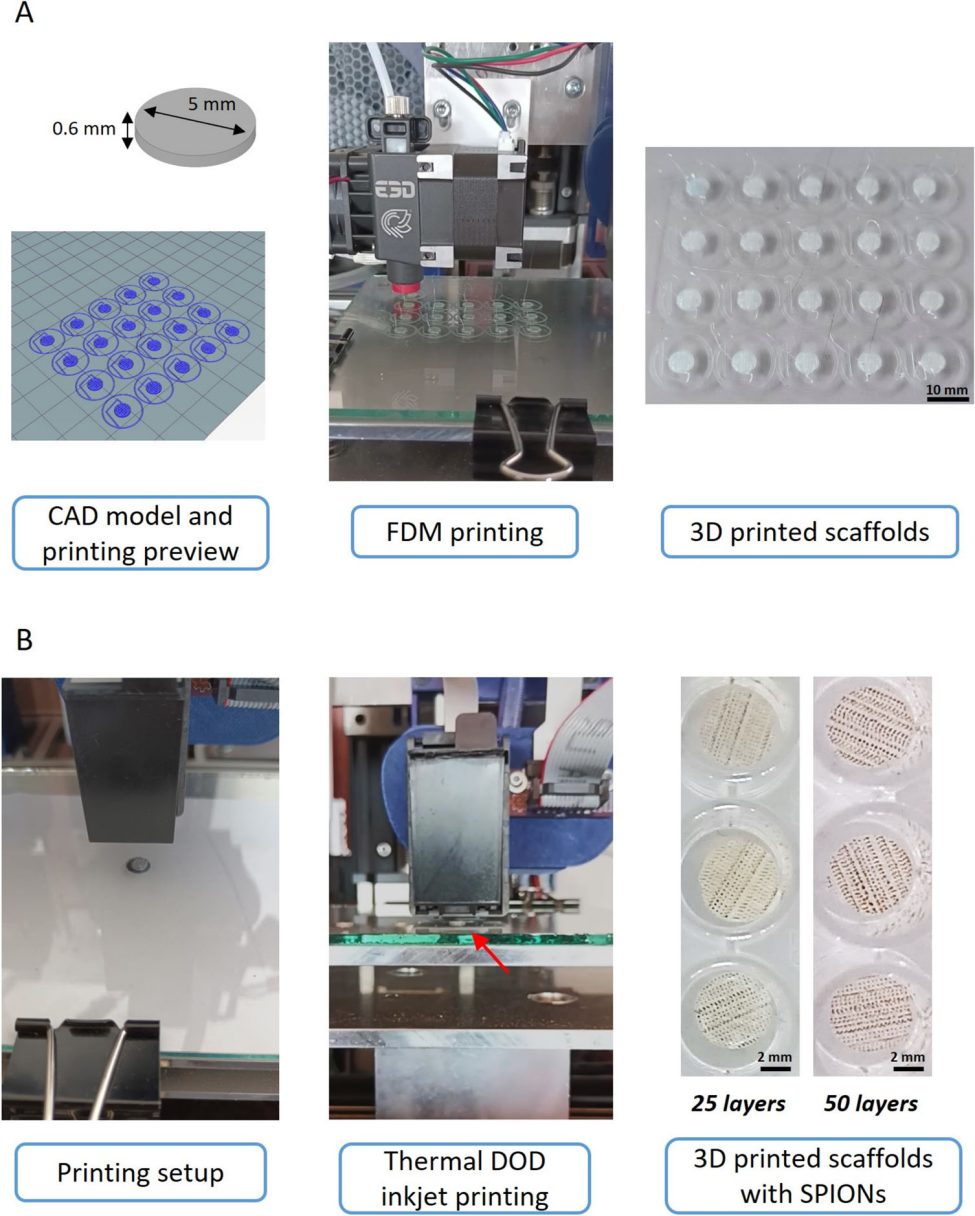
**A** FDM printing of cylindrical scaffold: CAD model and printing preview, FDM printing with the 3-P blend filament, and 3D printed scaffolds; **B** Inkjet printing SPIONs over the scaffold outer surface: Printing setup, SPIONs deposition on scaffold outer surface (pointed by the red arrow), and 3D printed scaffolds with 25 layers and 50 layers of SPIONs deposited over the surface

Scaffolds were 3D printed with a 3-polymer (3-P) blend filament composed of PLLA/PCL/PHBV (90/5/5 wt%). Biocompatibility and *in vitro* osteogenic potential of this polymer blend was previously reported [31, 32]. The polymer blend was prepared following an established protocol [31, 40]. and presented a filament diameter of 1.75 ± 0.2 mm. Printing parameters (*e.g.*, printing speed, nozzle, and bed temperature) were specifically optimized to print the 3-P blend filament with the printing platform through preliminary printing tests, starting from those reported in a previous study (Table 1) [32, 40]. To speed up the process, 20 scaffolds were 3D printed at a time on a glass slide (Fig. 1A). After printing, each scaffold diameter and height were measured using a digital caliper to assess the printing accuracy.

**Table 1:** Main printing parameters for 3D printing the cylindrical scaffolds and for the thermal DOD inkjet printing of SPIONs-loaded solutions over the 3D printed scaffold outer surface

3D printing				
Extrusion temperature [°C]	Bed temperature [°C]	Printing speed [mm/s]	Layer height [mm]	Infill [−]
190	40	10	0.2	100%

Thermal DOD inkjet printing				
Pulse voltage [V]	Firing pulse period [µs]	Firing delay period [µs]	Z distance [mm]	Printing speed [mm/s]
19	5	800	5	2

#### SPIONs thermal DOD inkjet printing

The thermal DOD inkjet printing module was used to deposit SPIONs-loaded droplets over the FDM-printed scaffold outer surface (Fig. 1B). Two different SPIONs-loaded solutions were tested: (i) DMSA-coated SPIONs colloidal suspension (SPIONs-DMSA) and (ii) colloidal suspensions of SPIONs functionalised with an RGD peptide (SPIONs-DMSA-RGD), both at 4 mg/mL concentration of SPIONs.

Before the inkjet printing of SPIONs-loaded solutions, the thermal DOD inkjet cartridges were thoroughly washed with deionized water to remove any presence of previous ink from the reservoir and nozzles. Then, the cartridge ink chamber was filled with 150 µL of SPIONs-loaded solution and mounted on the respective printer toolhead.

The 2D pattern of the ink was designed as a circle of 6 mm diameter to completely cover the scaffold's outer surface (diameter of 5 mm). The pattern resolution was 96 × 96 DPI as that of the inkjet cartridge. The pattern was exported as a grayscale binary image (*.bmp* format) to slice it with a previously developed software for inkjet image processing [35]. Specifically, the*.bmp* image is sliced from left to right in a zig-zag pattern and then converted to a 2D G-code file with the specific M command to send the firing pulse to specific nozzles according to the sliced image.

Printing parameters were optimized through preliminary tests as well as results reported from previous studies [41]. Specific distance values were defined along the X and Y axes of the printing plate to have dots equally distributed on the plane without overlapping. Dots were deposited on each scaffold at a distance between the cartridge and scaffold surface of 5 mm, and with a printing speed of 2 mm/s to avoid the formation of satellite drops. Each nozzle was activated by sending a pulse signal having a square wave characterized by a peak at 19 V for 6 µs (namely, T_on_), a delay of 800 µs (namely, T_off_) between two consecutive pulses on a given nozzle, and a delay of 0.5 µs before firing a different nozzle (Table 1). The 2D pattern was repeatedly inkjet on the same scaffold outer surface 25 and 50 times, for each of the two different SPIONs-loaded solutions, giving a total of 60 scaffolds per condition (Fig. 1B). From now on, polymer scaffolds without nanoparticles will be referred to as **SC**, scaffolds with 25 printing layers of SPIONs-DMSA as DMSA 25L, scaffolds with 25 printing layers of SPIONs-DMSA-RGD as RGD 25L, scaffolds with 50 printing layers of SPIONs-DMSA as DMSA 50L and scaffolds with 50 printing layers of SPIONs-DMSA-RGD as RGD 50L. In this case, the inject printing was performed on one side only, i.e. on the same side as the cell seeding in order to study the direct effect on the cellular response (biocompatibility, osteogenic differentiation), in the absence and presence of an external magnetic field.

### *In vitro* cell assays

Prior to cell assays, the scaffolds were quickly rinsed with Milli-Q water, sterilised in 70% ethanol solution and placed in 96-well plates. Human bone marrow-derived mesenchymal stem cells (hBM-MSCs) (Lonza, donor 38,157, passage 5) were cultured in mesenchymal stem cell growth medium (MSCBM supplemented with MCGS, GA-1000 and *L*-glutamine). Cells were washed with PBS and then trypsinised with 3 mL of 0.25% trypsin/EDTA. The cells were then centrifuged at 1000 rpm for 5 min. The resulting pellet was suspended in 1 mL of culture medium, the cell number was measured and the appropriate cell density for each different assay was seeded on the surface of the scaffolds. A seeding volume of 20 µl was used to achieve a droplet on the scaffold surface and thus cell adhesion to the scaffold surface; 2 h later, the wells were filled with 200 µl of culture medium. The cells were incubated at 37 °C in 5% CO_2_ for 24 h to allow cell adhesion. Controls were cells grown at the same cell density on the surface of similar polymeric scaffolds but without SPIONs on the surface.

#### *In vitro* biocompatibility assays

Biocompatibility assays were carried out with mesenchymal stem cell growth medium and the cell density used for seeding was 4 × 10^4^ cells/scaffold. The cells were cultured for 14 days on the surface of the scaffolds to assess their cytocompatibility. Cell proliferation was assessed at 4, 7, 10 and 14 days and cell adhesion at days 7 and 14. Cell viability and intracellular reactive oxygen species (ROS) content were assessed on day 14 by flow cytometry. Cells intended for assays analysed by flow cytometry were washed with 1 × PBS, collected with 0.25% trypsin/EDTA and processed for analysis.

##### Cell proliferation

Proliferation was measured using the WST-8 cell proliferation kit by obtaining absorbance values at 450 nm (colorimetric assay). The WST-8 cell proliferation kit is used to determine the number of viable cells and to study the induction or inhibition of cell proliferation *in vitro*. This assay kit is based on the cell reduction of the tetrazolium salt WST-8 (2-(2-methoxy-4-nitrophenyl)-3-(4-nitrophenyl)-5-(2,4-disulfophenyl)-2H-tetrazolium, monosodium salt) in a highly water-soluble, orange-coloured formazan dye.

##### Cell viability

Cell viability was measured by adding 0.005% (wt/vol) propidium iodide (PI) in PBS to the samples to stain the DNA of dead cells. PI exclusion indicates plasma membrane integrity and cell viability, as cells with plasma membrane damage incorporate the probe and the PI intercalates with their DNA. PI fluorescence was excited at 488 nm, and the emitted fluorescence was recorded with a 585/42 filter in a FACScalibur Becton Dickinson flow cytometer.

##### Intracellular ROS content

Cells were incubated with H_2_DCFDA 10 μM for 45 min at 37 °C. Non-fluorescent H_2_DCFDA is converted into 2′,7′-dichlorofluorescein (DCF) after hydrolysis by cellular esterases and oxidation by ROS. When DCF is excited at emission wavelengths of 488 nm, green fluorescence is emitted which can be detected at 525 nm. DCF fluorescence was measured in a FACScalibur Becton Dickinson flow cytometer with a 530/30 filter, exciting the sample at 488 nm.

##### Fluorescence confocal microscopy

Adhesion, colonisation, and morphology of cells on the scaffolds were studied by fluorescence microscopy after 7 and 14 days of culture. For confocal microscopy, cells attached to the scaffolds were washed twice with PBS and fixed with 4% (w/v) paraformaldehyde in PBS with 1% (w/v) sucrose at 37 °C for 20 min, washed again with PBS and permeabilised with 0.5% Triton X-100 at 4 °C for 5 min. The samples were then incubated at 37 °C for 20 min with Phalloidin-Atto 565 (1:40 dilution), which stains actin filaments. After washing with PBS, cell nuclei were stained with DAPI for 5 min. Confocal microscopy was analysed with an OLYMPUS FV1200 laser confocal microscope (OLYMPUS, Tokyo, Japan). Images were obtained using Imaris 3D software to project a single 2D image converted to a TIF file from the multiple Z-axis sections. DAPI and Phalloidin-Atto 565 staining were shown in blue and red, respectively.

##### Scanning electron microscopy

Adhesion, colonisation, and morphology of cells on the scaffolds were studied by SEM (JEOL JSM IT700HR) after 14 days of culture. Cells attached to the scaffolds were washed with PBS, fixed with a solution of 4% v/v paraformaldehyde and 2.5% v/v glutaraldehyde in milloning buffer at 4 °C for 4 h, then washed twice with distilled water and dehydrated in increasing concentrations (from 30 to 100% v/v) of ethanol. The scaffolds with cells on the surface were then dried in an automated critical point dryer (Leica EM CPD300), sputter-coated with a graphite layer in a turbo metalliser (sputter Quorum Q150 TE) and observed under a microscope at an accelerating voltage of 5 kV. To detach the cell monolayer and observe the scaffold surface, several areas on the surface of the samples were mechanically poked with tweezers prior to observation.

#### Osteogenic differentiation tests

As a proof of concept, preliminary osteogenic differentiation assays were performed with mesenchymal stem cell osteogenic differentiation medium and the cell density used for seeding was 6 × 10^4^ cells/well. 24 h after seeding, the culture plates were subjected to daily magnetic stimulation for 1 h and 1 Hz frequency using an oscillating magnetic force bioreactor (MICA Biosystems). Magnetic stimulation was applied for 14 days and markers of osteogenic differentiation were assessed on days 7 and 14. Plates were evaluated with the same study conditions but without applying magnetic stimulus as a control.

##### Gene expression by PCR

Total cellular RNA was isolated using a standard procedure (Trizol, Invitrogen, Carlsbad, CA, USA), and cDNA synthesis was performed using a high-capacity RNA-to-cDNA kit (Applied Biosystems, Thermo Fisher Scientific, Foster City, CA, USA). The expression levels of *ALP* and *Runx2* were determined by qRT-PCR using the Taqman probes Hs01029144_m1 and Hs01047973_m1 (FAM™ dye-labeled TaqMan® MGB probes, Applied Biosystems). For quantification of gene expression, the target gene values were normalized to the expression of the endogenous reference *GAPDH* (Glyceraldehyde 3-phosphate dehydrogenase, Hs02786624_g1). The comparative threshold cycle (Ct) method was used to calculate the relative expression. For quantification of gene expression, the target gene value was normalized to the expression of the endogenous reference. The amount of target, normalized to the endogenous reference and relative to the control is given by 2^−ΔΔCt^ (Relative Quantification, RQ). (ΔCt = Ct _target gene_—Ct _endogenous reference_; ΔΔCt = ΔCt _study condition_—ΔCt _control_) [42].

##### ALP enzymatic activity

ALP activity was used as a key differentiation marker to assess the expression of the osteoblastic phenotype. Cells were lysed by three consecutive freeze–thaw cycles. The lysates were incubated with a 10 mM *p*-nitrophenylphosphate solution and then the reactions were stopped by adding 2 M NaOH. Cellular ALP activities were measured spectrophotometrically, by measuring the increase in absorbance at 405 nm accompanying the production of p-nitrophenol and normalized by the cell protein content, which was determined using a total protein quantitative kit.

##### Mineralisation

Matrix mineralisation was analysed by alizarin red S staining. Cells were washed with PBS twice, fixed with 70% ethanol for 1 h at 4 °C and stained with 40 mM alizarin red S for 30 min. After this time, cells were washed thoroughly with distilled H_2_O to remove non-specific staining. Finally, to quantify matrix mineralisation, the stained samples were eluted with cetylpyridinium chloride (10% w/v) in 10 mM PBS pH 7 and the absorbance was measured at 620 nm.

##### Osteocalcin secretion

The amount of osteocalcin secreted into the supernatant by hBM-MSCs cultured on the scaffolds was measured using a Human Osteocalcin (OC) ELISA Kit according to the manufacturer's instructions (Bioss ANTIBODIES). The concentration of OC in the samples was determined by comparing the O.D. measured at 450 nm of the samples to the standard curve. The osteocalcin content was expressed as pg/mL.

## Results

### Preparation of colloidal SPIONs suspensions

SPIONs were prepared by exploiting the method of thermal decomposition of iron (III) oleate as an iron-containing precursor [33]. After synthesis, structural characterisation of the oleic acid-stabilised nanoparticles (SPIONs-OA) was performed. The XRD pattern (Fig. S2A) showed multiple peaks consistent with the inverse spinel structure of magnetite [43] and TEM analysis (Fig. S2B) confirmed a uniform nanoparticle size, with dimensions of about 11 nm. The nanoparticles were further processed by exploiting a ligand exchange of the oleic acid surface layer with DMSA to obtain a stable suspension of nanoparticles in water (SPIONs-DMSA) [15, 33]. The FTIR spectrum (Fig. S4A) confirmed the success of the ligand exchange. Next, SPIONs-DMSA nanoparticles were covalently functionalised with an Arg-Gly-Asp (RGD) peptide via amide bonds to obtain the SPIONs-DMSA-RGD suspension (Fig. S3). For this purpose, the carboxylic acid groups present in the DMSA coating of the SPIONs were activated through EDC/sulfoNHS chemistry and reacted with the primary amine of the RGD sequence. The functionalisation was verified by FTIR, DLS (Fig. S4A-B) and chemical microanalysis (Table S1) [34]. The magnetization curves of the synthesised SPIONs showed absence of hysteresis loop, therefore confirming their superparamagnetic behaviour (Fig. S2C and S4C).

### Scaffold design and fabrication

The combination of the FDM printing and thermal DOD inkjet of SPIONs was achieved thanks to the calibrated and accurate printing platform (*see *Supplementary Video S1*.mp4 and Video S2.mp4*). Scaffolds were 3D printed with a 3-polymer (3-P) blend filament composed of PLLA/PCL/PHBV (90/5/5 wt%). Biocompatibility and *in vitro* osteogenic potential of this polymer blend was previously reported [31, 32]. Each of the printed scaffolds (n = 300) was manually measured with a digital calliper in diameter and height, giving an average diameter and height of, respectively, 4.98 mm and 0.63 mm, and thus, a relative error of 0.4% and 5%. The accuracy (computed as the standard deviation of the difference between the correct diameter/height, 5 mm/0.6 mm), was ± 60 µm in diameter and ± 20 µm in height. Globally, it can be stated that the accuracy of 3D printed scaffolds was ± 40 µm whereas, for comparison, that of commercial FDM printers is ± 100 µm [44].

Two different SPIONs solutions were tested for the thermal DOD inkjet printing: *(i)* colloidal suspension of DMSA-coated SPIONs (SPIONs-DMSA) and *(ii)* colloidal suspension of SPIONs functionalised with an RGD peptide (SPIONs-DMSA-RGD). Currently, our research group have demonstrated the efficacy of these free-magnetic nanoparticles when exposed to the application of an external magnetic field for osteogenic purposes in hBM-MSCs. The results derived from this research indicates that remotely activated mechanotransduction using integrin-targeted magnetic colloidal SPIONs provokes a significant increase in runt-related transcription factor 2 (Runx2) and alkaline phosphatase (ALP) gene expression as well as ALP activity compared with non-targeted SPIONs (SPIONs-DMSA). In fact, a significant osteogenic differentiation is observed when the magnetic stimulus is applied when the nanoparticles encounter the cell membrane surface to initiate endocytic pathways. Then, in this manuscript both types of nanoparticles have been incorporated into the FDM devices to study the possible effect of integrin-targeting functionalisation on nanoparticles once immobilized in the scaffolds. The 2D pattern was repeatedly inkjet on the same scaffold outer surface 25 and 50 times, for each of the two different SPIONs-loaded solutions. From now on, polymer scaffolds without nanoparticles will be referred to as SC, scaffolds with 25 printing layers of SPIONs-DMSA as DMSA 25L, scaffolds with 25 printing layers of SPIONs-DMSA-RGD as RGD 25L, scaffolds with 50 printing layers of SPIONs-DMSA as DMSA 50L and scaffolds with 50 printing layers of SPIONs-DMSA-RGD as RGD 50L.

### Characterisation of fabricated scaffolds

**Fig. 2:**
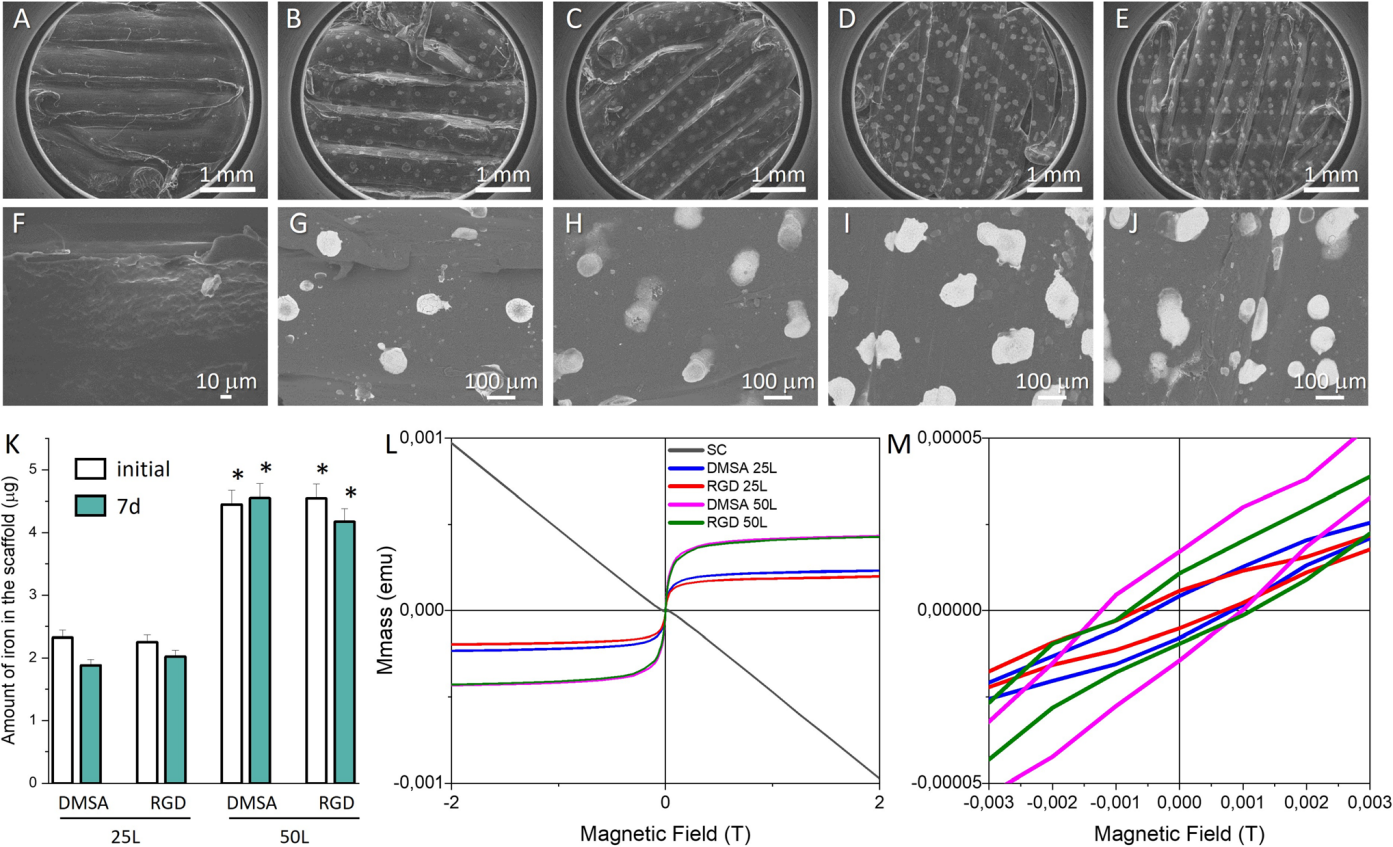
**A**, F SEM images corresponding to SC; **B**, G SEM images corresponding to DMSA; **C**, H SEM images corresponding to DMSA 25L; **D**, **I** SEM images corresponding to DMSA 50L; and **E**, **J** SEM images corresponding to 50L, showing their surface. **A**–**E** Images at low magnification. **F**–**J** SEM images at higher magnification. These SEM studies have been carried out in a microscope field emission JSM6335 with a back scattering electron detector. **K** Stability of the different scaffolds with SPIONs showing the initial amount of Fe and the amount of Fe remaining in the scaffolds after 7 days in 1 × PBS at 37 °C. Asterisks indicate significant differences with respect to 25L (*p* value < 0.05). **L** Magnetisation curves of the different scaffolds. **M** Magnification of the curves at low fields

The morphology of the printed scaffolds was evaluated by SEM (Fig. 2A-E), which showed a generally smooth surface of the material with areas of increased roughness at the edge between one printed line and another. The filament width of the scaffolds was measured with ImageJ software [45] which corresponded to 0.58 ± 0.03 µm (average over n = 4 3D printed scaffolds). Filaments appeared well stacked with no visible pores on the surface, as expected (infill equal to 100%, Table 1). At low magnification (Fig. 2B-E), the achievement of the intended inkjet 2D tetragonal pattern was observed in the circular scaffold surface. At higher magnification in the SEM images (Fig. 2G-J), homogeneous deposits of SPIONs following the 2D pattern was observed, with neatly distributed spots over the entire circular surface. The "brighter" SEM intensity correlates with a higher average atomic number in the sample, and "dark" areas have a lower average atomic number. In this case it is easy to attribute the areas of higher brightness in the images to deposits of SPIONs due to the difference between the atomic number of iron and carbon. To determine the differences between the samples a study by high resolution scanning microscopy using a JSM7600 microscope coupled to an EDS detector was carried out (Fig. S6). The EDS-mapping revealed the homogeneous distribution of iron (in red) which is concentrated in the spots resulting from the DOD process. There were some iron satellites outside the spots nonetheless being almost negligible. HRSEM studies showed also a very homogeneous size of the iron spots with slight increase in size for 50L samples.

Figure 2K shows the amount of iron on the surface of the scaffolds after production (initial), and the amount of iron remaining in the scaffolds after 7 days in physiological media, measured by ICP-AES. The results obtained for DMSA 25L and RGD 25L were 2.32 and 2.25 µg iron, respectively. The resulting amounts for DMSA 50L and RGD 50L were 4.45 and 4.54 µg respectively. The amount of SPIONs deposited was doubled by doubling the number of printed layers, indicating a high accuracy in the production technique and versatility in producing new materials with different amount of SPIONs. Furthermore, no significant differences were observed in the amount of SPIONs deposited between the SPIONs-DMSA and SPIONs-DMSA-RGD suspensions for the same number of layers. Likewise, no significant differences were observed in the amount of iron on the surface of the scaffolds after 7 days in 1 × PBS at 37 °C with respect to the initial content, indicating that the SPIONs deposits are very stable after soaking and remain on the surface.

The evaluation of the magnetic properties of the materials showed a diamagnetic behaviour (negative slope) of the control scaffold (SC, scaffold without SPIONs), therefore, initially in the magnetisation curves of the scaffolds with SPIONs two components were observed, a diamagnetic one attributed to the polymer scaffold and a superparamagnetic one attributed to the SPIONs. The magnetisation curves of the DMSA 25L, RGD 25L, DMSA 50L and RGD 50L scaffolds (Fig. 2L) were plot after correction of the diamagnetic component. The curves corresponding to the scaffolds with 50 printed layers of SPIONs showed a saturation magnetisation value double that observed in the curves corresponding to the scaffolds with 25 printed layers. This increase is due to the amount of SPIONs present in the scaffold, in line with the ICP-AES result and SEM–EDS studies (Fig. 2K) where approximately twice as much iron is observed in the scaffolds with 50 printed layers. With respect to the type of nanoparticle incorporated (SPIONs-DMSA or SPIONs-DMSA-RGD), no major differences are detected. When the scaffolds have 50 printed layers the curves are similar (DMSA 50L and RGD 50L), and when they have 25 printed layers, the magnetisation of DMSA 25L is slightly higher than that of RGD 25L, which could be due to a slight difference in the amount of SPIONs between the samples, but still the values are very similar. Furthermore, the magnetisation curves of both SPIONs alone and incorporated on the scaffolds showed similar shape and coercivity. As shown in the graphs, in both cases coercivity values below 3 mT were recorded, which for most biomedical applications of SPIONs can be considered as a non-hysteretic response, *i.e.*, coercivity and remanence close to zero; confirming their superparamagnetic behaviour (see Fig. 2M and insets of Fig. S2C, S4C and S5). Finally, the maximum magnetisation values normalised to the Fe content calculated for the SPIONs alone (Fig. S4C) and the scaffolds with SPIONs (Fig. S5) were consistent, obtaining a value around 90 emu/g_Fe_ in all cases. Therefore, the similarity between the different cycles suggests that the magnetic properties of the SPIONs were not affected by their incorporation on the surface of the polymer scaffolds.

**Fig. 3:**
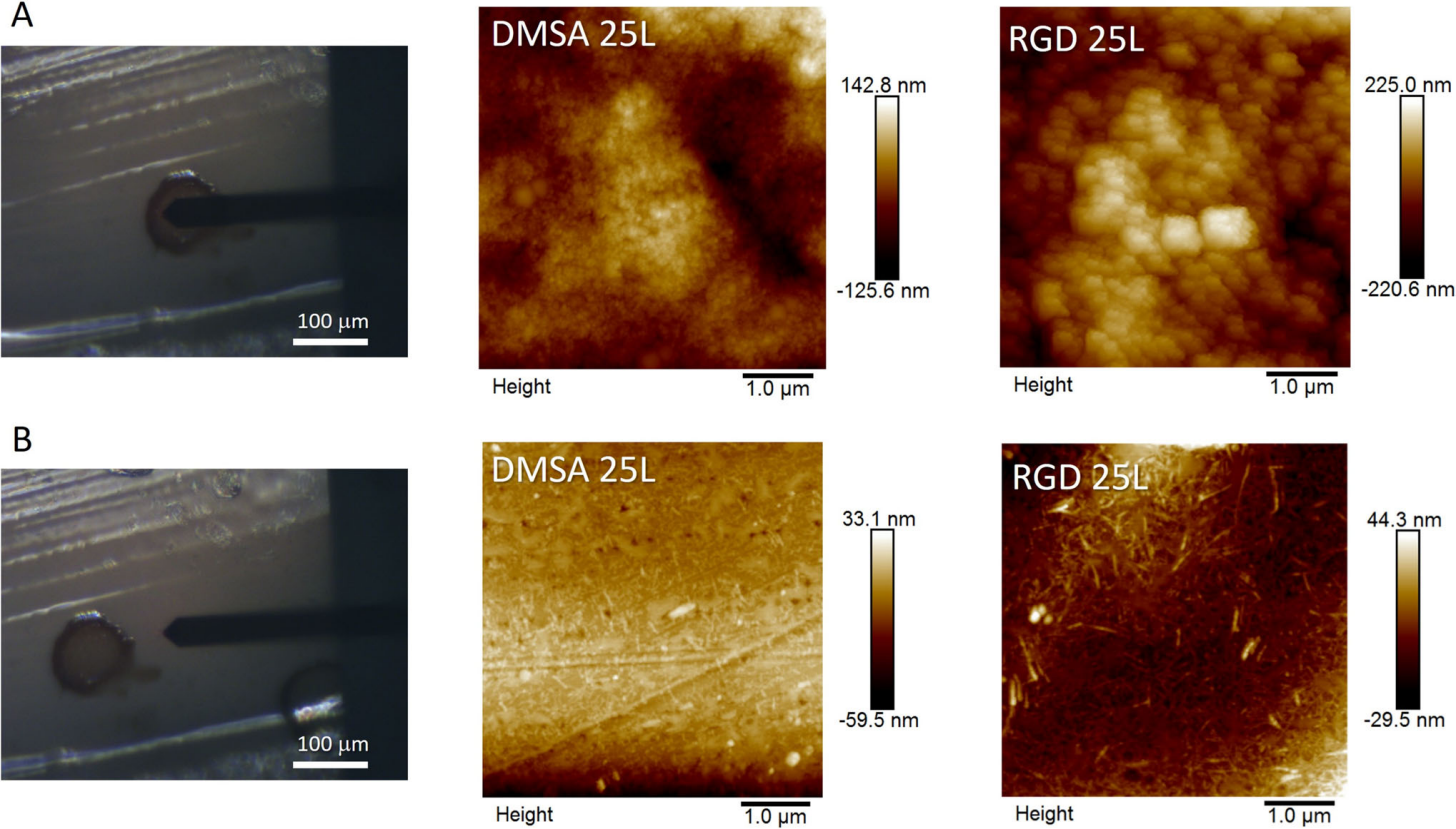
AFM images of the DMSA 25L and RGD 25L samples showing their surfaces. For all samples, an area of 5 µm × 5 µm was scanned. The photographic images of the camera attached to the AFM equipment (images **A** and **B** on the left) indicate the area where the measurement was taken. Images **A** centre and right: analysis of an area with SPIONs (within the SPIONs spots). Images **B** centre and right: analysis of an area without SPIONs (outside the SPIONs spots, i.e. on the polymeric surface of the scaffold)

Finally, to obtain more information at nanoscale, a deep AFM study was carried out. The most significant results are shown in Fig. 3 and Figure S7. Two different areas of the scaffolds have been studied (as indicated by the position of the AFM cantilever in the photographic images in Fig. 3 left), one inside a magnetic spot (Fig. 3A) and one outside (on the polymeric surface of the scaffold, Fig. 3B). A scan of 5 µm × 5 µm has been carried out in all areas studied showing notables differences between the different areas. While in the interior of the spots an agglomerate of nanoparticles is observed with an increase in z-height, in the area outside, a quasi-smooth zone is observed, typical of the polymeric surface of the FDM scaffold. The roughness value (Ra) for the pristine FDM scaffolds is of 5.2 nm in the measured area while Ra values within the spots is increased with a value of 29.9 nm and 52.5 nm for the DMSA 25L and RGD 25L samples, respectively. Note that the Ra values measured in the outside area are in agreement with those obtained for the control sample (8.3 nm and 7.9 nm for DMSA-25L and RGD-25L). This slight increase could be due to the incipient deposition of nanoparticles outside the spot area observed by EDS mapping (Fig. S6). The increase in the Ra value in the zone inside the spot is due to the deposition of the nanoparticles (which are perfectly visible in the AFM images), which is greatest with the degree of aggregation of the starting nanoparticles described above [34].

### *In vitro* biocompatibility

**Fig. 4:**
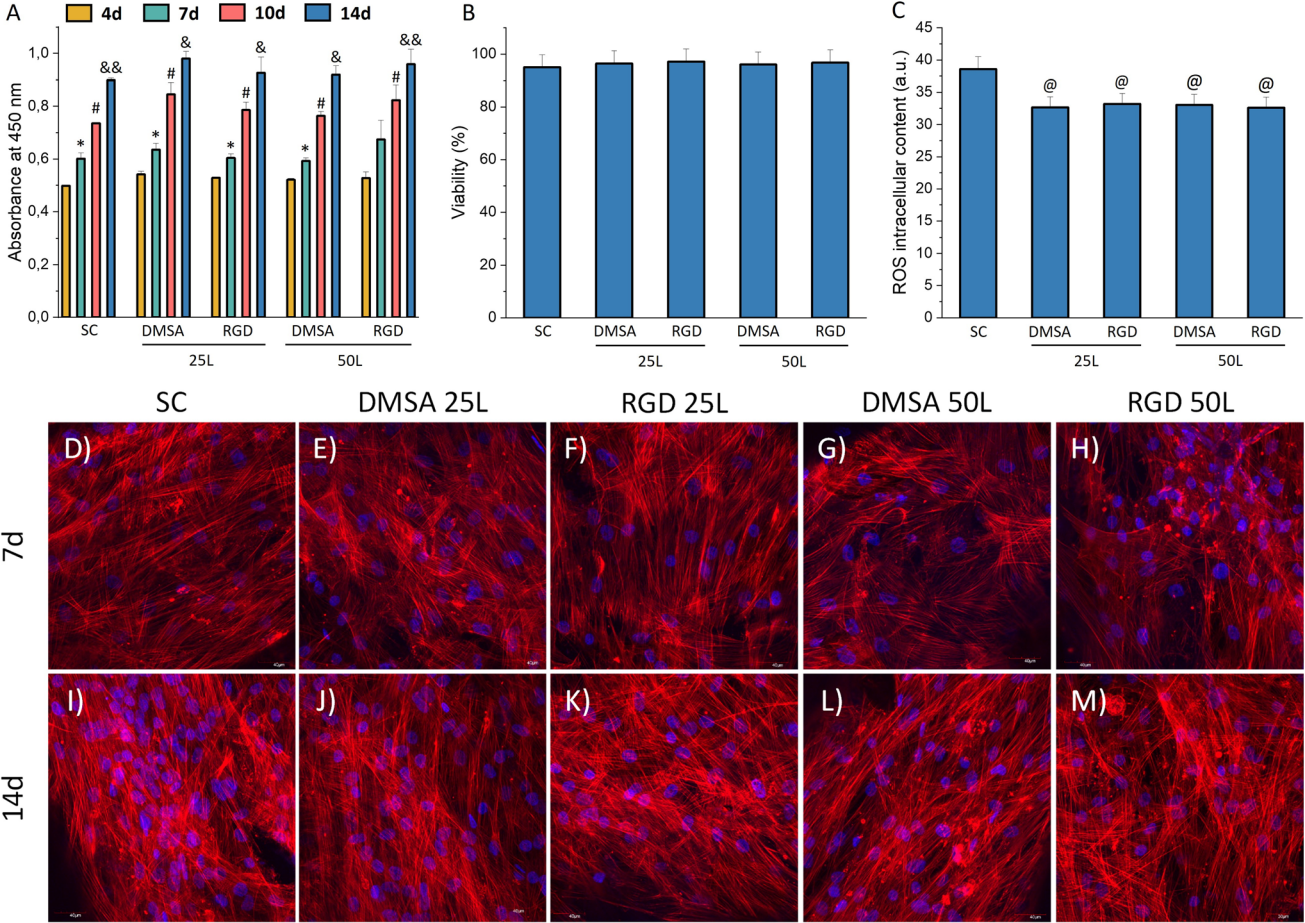
**A** Proliferation at 4, 7, 10 and 14 days, **B** viability at 14 days and **C** intracellular ROS content at 14 days of hBM-MSCs cultured on SC, DMSA 25L, RGD 25L, DMSA 50L and RGD 50L. Asterisks indicate significant differences with respect to 4d (* *p*-value < 0.05). Hashtags indicate significant differences with respect to 7d (# *p*-value < 0.05). Ampersands indicate significant differences with respect to 10d (& *p*-value < 0.05 && *p*-value < 0.01). At symbols indicate significant differences with respect to SC (@ *p*-value < 0.05). **D**–**M** Confocal fluorescence microscopy images of hBM-MSCs cultured on the different scaffolds at 7 and 14 days. In red can be seen the actin filaments of the cytoskeleton labelled with phalloidin and in blue the nuclei labelled with DAPI

Cell adhesion, viability and proliferation are important biological parameters to assess the biocompatibility or cytotoxicity of any biomaterial. Cell adhesion to biomaterials is crucial for downstream cellular processes to occur. Cell proliferation, the process by which a cell grows and divides into two daughter cells, reflects the growth capacity of the cells in contact with the materials to be tested. Cell viability is a parameter related to the integrity of the cell membrane. Thus, adequate cell adhesion accompanied by maintenance of cell viability and allowing cell proliferation is a desired outcome to validate a biomaterial with potential for tissue regeneration. The proliferation of hBM-MSCs in contact with the scaffolds surface was studied at 4, 7, 10 and 14 days (Fig. 4A). Results show that hBM-MSCs are able to proliferate on the surface of the different scaffolds over time. No differences in cell proliferation were observed between the control scaffold and the scaffolds with SPIONs at any of the times studied. Regarding the viability of hBM-MSCs (Fig. 4B), the results indicated very high viability percentages in all cases studied (SC, DMSA 25L, RGD 25L, DMSA 50L and RGD 50L), with no significant differences between them (≥ 95% viability).

Several studies have proposed oxidative stress as a key mechanism involved in nanomaterials toxicity [46]. Overproduction of ROS leads to multiple adverse biological effects such as membrane lipid peroxidation, protein denaturation, mitochondrial dysfunction, lactate dehydrogenase leakage, and DNA and RNA damage, limiting the cellular ability to maintain normal physiological redox-regulated functions [47]. Moreover, several studies have highlighted the importance of measuring cellular ROS production in response to biomaterial surfaces to assess their *in vitro* biocompatibility [48]. In order to study possible oxidative stress in hBM-MSCs after interaction with the scaffolds surface, the intracellular ROS content was assessed after 14 days of culture (Fig. 4C). A decrease in intracellular ROS content was observed in cells cultured on the surface of scaffolds with SPIONs deposits compared to the control scaffold. These results are in agreement with other studies analysing the effect of SPIONs on hBM-MSCs and neural stem cells (NSCs) [14, 49], where contact with SPIONs also decreases the intracellular ROS content.

Adhesion, colonisation and cell morphology of hBM-MSCs cultured on the surface of the scaffolds were assessed by laser scanning confocal microscopy (Fig. 4D-M) and scanning electron microscopy (Fig. S8). Different parts of the scaffolds were scanned after 7 and 14 days of culture, using Phalloidin-Atto 565 as fluorescence probe for F-actin microfilaments and DAPI for nuclei. The images showed that hBM-MSCs presented their typical spindle-shaped morphology, adhered perfectly to the surface of the scaffolds and colonised the entire surface without leaving gaps. No differences were observed between the scaffolds with SPIONs and the control scaffold, neither between the type of SPIONs (DMSA or RGD) deposited, nor in the number of printed layers (25L or 50L). In addition, a higher number of cells is discernible in the 14-day images compared to the 7-day images, which is consistent with the cell proliferation results. The results showed a complete colonisation forming an adequate cellular lattice regardless of the presence of SPIONs. This result is confirmed by SEM images (Fig. S8).

In addition, another SEM analysis of the scaffolds colonised by the hBM-MSCs was carried out. In this case, prior to observation, several areas of the surface of the samples were punctured with tweezers to detach the cell monolayer and expose the surface of the scaffold. The images showed deposits of SPIONs under the cell monolayer (Figure S9), confirming their permanence on the scaffold surface after 14 days of culture with hBM-MSCs, thus ensuring long-term contact between the SPIONs and the cell surface.

Taken together all these *in vitro* studies show that the incorporation of the SPIONs in the scaffolds by the DOD technique does not affect their biocompatibility in the two conditions studied and by increasing the number of SPIONs printed layers on the scaffold. The cytocompatibility results of this work in terms of viability, proliferation and cell adhesion agree with the results provided by other authors in different studies with other types of scaffolds also incorporating SPIONs in their composition [50–52].

### Osteogenic effect

**Fig. 5:**
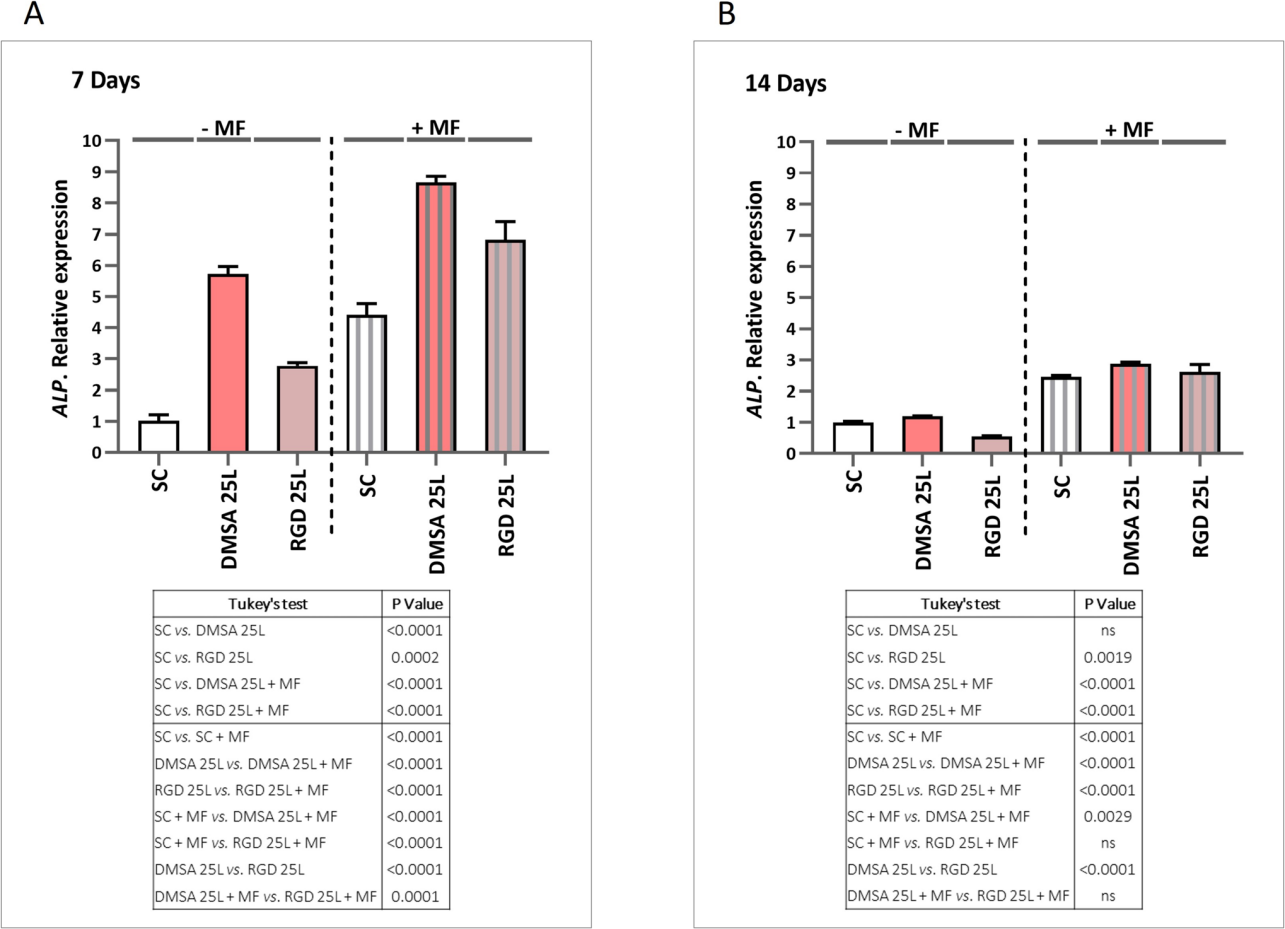
Evaluation of ALP gene expression in hBM-MSCs cultured on the surface of SC, DMSA 25L and RGD 25L scaffolds by applying (+ MF) and not applying (− MF) an external magnetic field of 1 Hz frequency for 1 h per day. **A** Expression was measured at 7 days and **B** 14 days. Results were statistically analysed by one-way ANOVA and Tukey's test

**Fig. 6:**
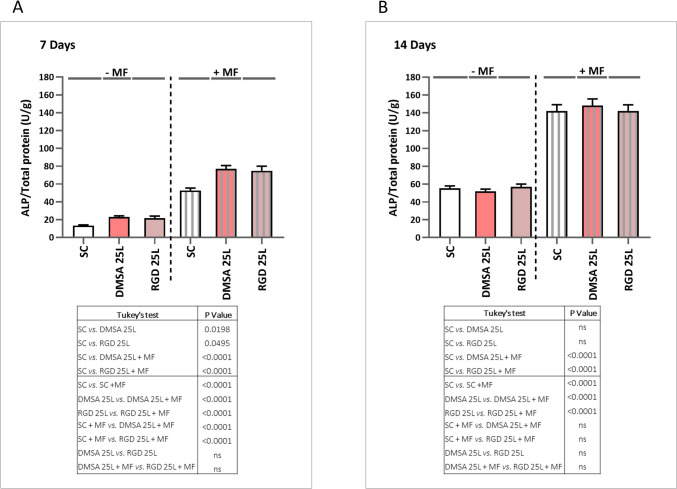
Evaluation of ALP activity in hBM-MSCs cultured on the surface of SC, DMSA 25L and RGD 25L scaffolds by applying (+ MF) and not applying (− MF) an external magnetic field of 1 Hz frequency for 1 h per day. **A** Expression was measured at 7 days and **B** 14 days. Results were statistically analysed by one-way ANOVA and Tukey’s test

**Fig. 7:**
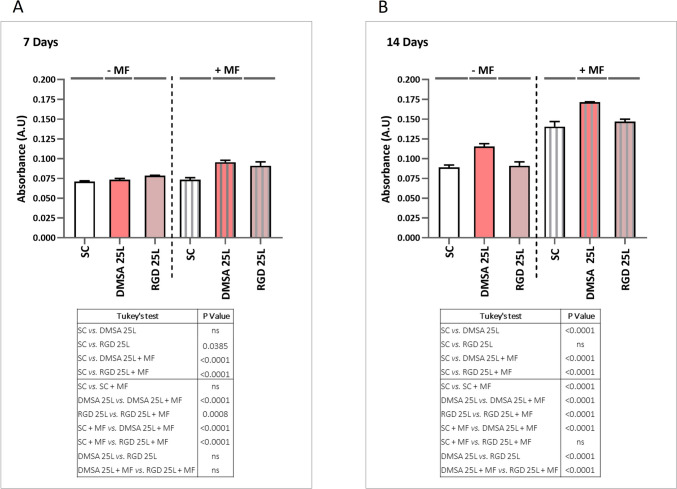
Evaluation of the mineralisation process in hBM-MSCs cultured on the surface of SC, DMSA 25L and RGD 25L scaffolds by applying (+ MF) and not applying (− MF) an external magnetic field of 1 Hz frequency for 1 h per day. **A** Expression was measured at 7 days and **B** 14 days. Calcium deposits stained with alizarin red were quantified by measuring absorbance at 620 nm. Results were statistically analysed by one-way ANOVA and Tukey’s test

ALP glycoprotein on the cell surface hydrolyses the mineralisation inhibitor pyrophosphate to phosphate, which promotes mineral deposition in ECM collagen fibres [53–55]. As ALP potentiates ECM mineralisation, quantification of ALP at both the mRNA and protein level (enzymatic activity) has been used to describe osteoblast differentiation [56]. Also, as matrix mineralisation represents the last step of differentiation, the measurement of this marker is a very important outcome to determine the efficacy of osteoblast differentiation in bone research [56]. Here, the osteogenic differentiation of hBM-MSCs was assessed by measuring *ALP* and *Runx2* expression level, ALP activity, mineralisation process and osteocalcin (OC) secretion. These markers were measured in the cells grown on the surface of the scaffolds with 25 printed layers of SPIONs, both in the presence and absence of the magnetic field. Our results revealed that 7 days of cell culture on the surface of both types of scaffolds with SPIONs deposits (DMSA 25L and RGD 25L) promoted a significant increase in *ALP* expression both in presence and absence of the magnetic field (MF), compared to cells cultured on the scaffolds without nanoparticles (SC and SC + MF respectively). After exposure of cells to MF, *ALP* gene expression was significantly increased in all conditions compared to unexposed cells (Fig. 5A). Thus, there was an increase of *ALP* expression associated exclusively with the material (SPIONs deposited on the scaffold surface), an increase associated exclusively with the magnetic field (SC + MF condition) and a larger increase associated with the combination of both (SPIONs deposited on the surface and + MF). Following the trend of gene expression at 7 days, a significant increase in ALP activity was observed in cells grown on DMSA 25L and RGD 25L scaffolds compared to cells grown on the control scaffolds (SC and SC + MF respectively), both applying and not applying MF. After exposure of cells to MF, ALP activity was significantly increased in all conditions compared to unexposed cells (Fig. 6A). Again, these results reveal one increase attributed to the presence of SPIONs, another related to MF, and the greatest increase when SPIONs are activated by MF. The results suggest that both types of scaffolds combined with the magnetic field promote efficient differentiation toward the osteogenic lineage of the human mesenchymal stem cells used in this study, as ALP expression has been described in preosteoblasts [56] and mature osteoblasts [57]. Our data agree with previous studies that have shown that SPIONs, static magnetic fields, and the combination of both promote osteogenic differentiation [34]. Since ALP potentiates extracellular matrix mineralisation [56, 58], which is another sign of osteogenic differentiation, we evaluated this process in our model. Figure 7A shows the results after 7 days of culture: despite being a very early time to evaluate a marker of osteogenic differentiation such as mineralisation, a significant increase in absorbance was observed for DMSA 25L + MF and RGD 25L + MF conditions compared to SC and SC + MF. This could suggest that in the DMSA 25L + MF and RGD 25L + MF scaffolds the cells start to mineralize earlier than in the other conditions, so that in a short time [7 days] the effect of osteogenic differentiation through the mechanotransduction process (MF-activated SPIONs) can be observed. Moreover, among the multiple transcription factors regulating osteoblast differentiation, RUNX2 is a master transcription factor in the differentiation of MSCs to osteoblasts [59]. Therefore, RUNX2 expression is commonly used as a marker of osteogenic differentiation of MSCs. RUNX2 is considered the master switch for the initiation of osteogenesis, as RUNX2 is expressed in MSCs and upregulated in pre-osteoblasts, while in mature osteoblasts, RUNX2 expression is downregulated [60, 61]. In this study we also measured the expression level of *Runx2* at time 7 days. The results show a significant decrease in *Runx2* expression in the DMSA 25L + MF and RGD 25L + MF conditions compared to the unstimulated conditions (DMSA 25L and RGD 25L respectively) (Fig. S10 in Supplementary Material). Furthermore, the lowest *Runx2* expression values occur in the magnetic field stimulated conditions (+ MF) coinciding with the conditions with the highest values of *ALP* expression, ALP enzyme activity and mineralisation. These results could suggest that cells cultured on the surface of scaffolds with SPIONs deposits and subjected to magnetic stimuli (DMSA 25L + MF and RGD 25L + MF) differentiate into osteoblasts and start the mineralisation phase earlier than in the other conditions.

**Fig. 8:**
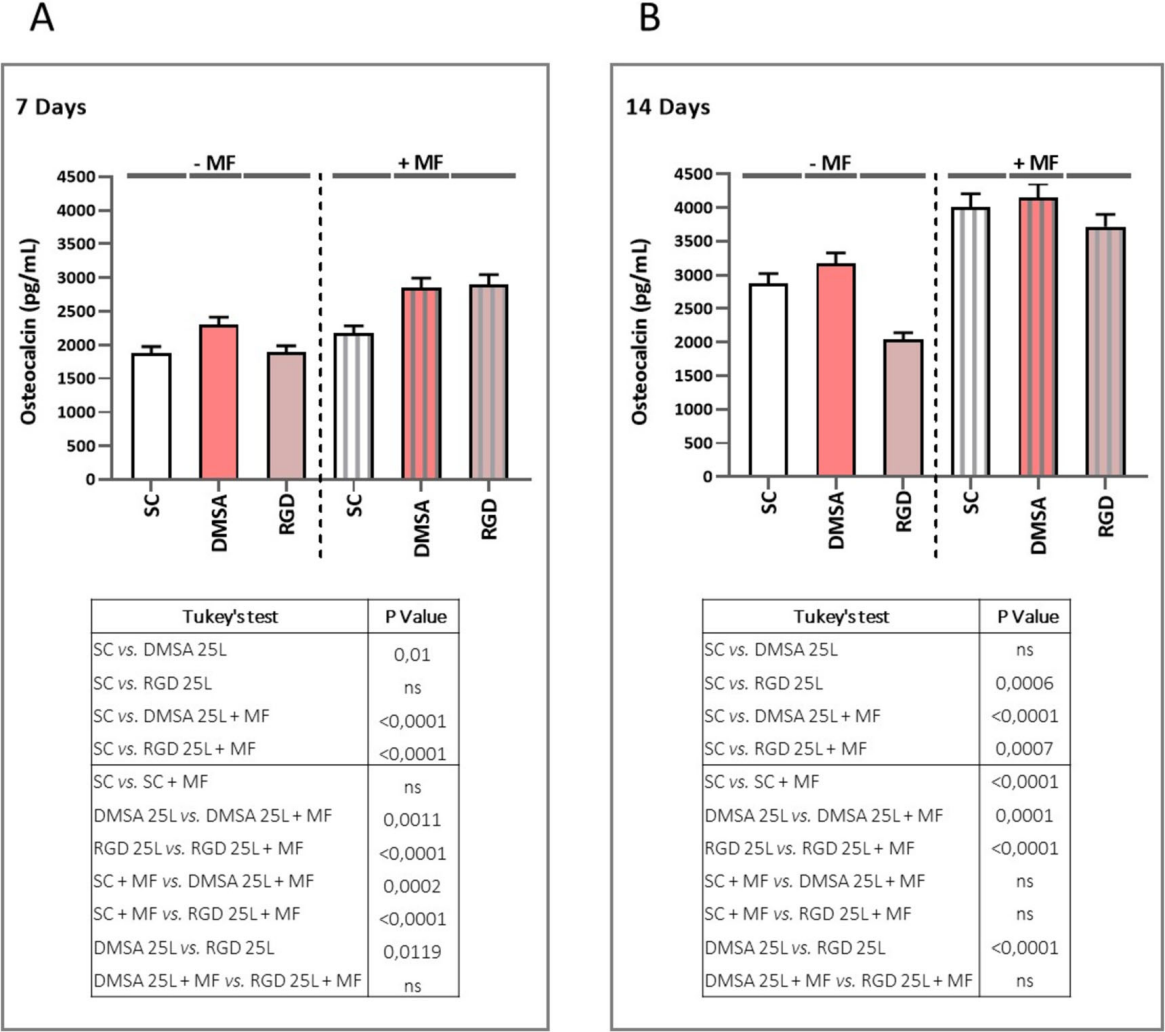
Evaluation of the amount of OC secreted by hBM-MSCs cultured on the surface of SC, DMSA 25L and RGD 25L scaffolds by applying (+ MF) and not applying (− MF) an external magnetic field of 1 Hz frequency for 1 h per day. **A** Amount of OC measured at 7 days and **B** at 14 days. Results were statistically analysed by one-way ANOVA and Tukey’s test

Considering 14 days of cell culture in the absence of MF, *ALP* gene expression was maintained in the DMSA 25L scaffold, *i.e.*, no significant differences were detected compared to the SC condition. However, a significant decrease in gene expression was detected in the RGD 25L scaffold compared to cells cultured in SC. Again, in the presence of MF, gene expression was significantly increased in all conditions compared to unexposed cells. The results suggest that at short times (7 days) it is possible to detect the mechanotransduction effect, because the increase in *ALP* gene expression in the DMSA 25L + MF and RGD 25L + MF conditions is significantly higher than the increase in the SC + MF condition. Nevertheless, at long times (14 days) such effect is not so evident, since no differences are observed between the SC + MF and RGD 25L + MF conditions, even though a significant increase is still observed in the DMSA 25L + MF condition with respect to SC + MF. Results are shown in Fig. 5 A and B, respectively. At 14 days (Fig. 6B), no significant differences in ALP activity were observed between DMSA 25L and RGD 25L conditions with respect to SC, neither applying nor not applying MF. After exposure of cells to MF, ALP activity was significantly increased in all conditions compared to unexposed cells. Again, this result resembles that obtained in the 14 day gene expression analysis. An increase in ALP activity associated with the magnetic field is observed, but no differences are observed between the scaffolds with SPIONs and the control scaffolds. This could be due to the fact that both, the osteogenic effect produced by the differentiation medium (osteogenic factors) and the osteogenic effect produced by the differentiation medium plus the magnetic field, for a prolonged time (14 days), are much higher than the effect associated with the SPIONs and the effect associated with the SPIONs activated by the MF. Therefore, the effect of the differentiation medium at long time (14 days) might be masking the effect due to mechanotransduction, but at short time (7 days) it is evident. Finally, at 14 days (Fig. 7B), an increase in mineralisation was observed in cells cultured on the DMSA 25L scaffold compared to cells on the scaffold without SPIONs both with and without applying magnetic field. Furthermore, in the presence of MF, mineralisation was significantly increased in all conditions compared to unexposed cells. These mineralisation results are in agreement with those obtained for osteocalcin secretion (Fig. 8). After 7 days of culture (Fig. 8A), a significant increase in the amount of OC secreted was observed in the DMSA 25L + MF and RGD 25L + MF conditions compared to SC and SC + MF, as observed in the mineralisation process. At 14 days (Fig. 8B), a significant increase in OC secreted by cells cultured on scaffolds where MF was applied was observed compared to unexposed cells. Results were statistically analysed by one-way ANOVA (*P* < 0.001) and Tukey's test.

Hu et al*.* [62] demonstrated enhanced bone regeneration *in vivo* in rats using a gelatine sponge (GS) loaded with SPIONs as a scaffold (SPIONs-GS). The results ensured that SPIONs induced active osteogenesis without using an external magnetic field. Jia et al*.* [63] coated 3D printed scaffolds with SPIONs and used for palate bone regeneration in a rat model. The results demonstrated that scaffolds coated with SPIONs can be used to treat defects of the palate. Moreover, several studies have revealed that static magnetic fields can regulate the proliferation, differentiation and function of bone tissue cells, including hBM-MSCs, osteoblasts, osteoclasts and osteocytes. As well as a large number of animal experiments and clinical studies have shown that static magnetic fields have effective therapeutic effects on bone-related diseases such as non-healing fractures, non-union of bone implants, osteoporosis and osteoarthritis [64]. In recent years, research on the combination of static magnetic fields with bone regenerative materials, especially magnetic materials, has increased [58]. Zhang et al*.* have also shown that the magnetic field stimulates osteogenic differentiation of MSCs on magnetic scaffolds [65]. However, unlike our results, they do not detect the osteogenic effect in the short term (7 days) and do detect it in the 14-day culture. This suggests that in their materials, magnetic particles coupled to the magnetic field could promote osteogenesis, but a longer exposure time is needed.

Regarding the presence or absence of RGD peptide in SPIONs deposited on the surface of the scaffolds, after 7 days of culture no significant differences were observed in either ALP activity or mineralisation, regardless of whether or not a magnetic field was applied (DMSA 25L vs. RGD 25L and DMSA 25L + MF vs. RGD 25L + MF). After 14 days of culture, there was also no difference between the presence or absence of RGD peptide on the ALP activity of the cells. Functionalisation of SPIONs with the RGD peptide was carried out with the aim of favouring interactions between SPIONs and cellular integrins, which can act as cellular mechanosensors [66–68]. The recent study by our group [34], demonstrates that functionalisation of this type of SPIONs with RGD peptide enhances osteogenic differentiation of hBM-MSCs when added in aqueous solution on cells and magnetic field is applied after the first 30 min upon incorporation. However, in the present strategy SPIONs are in the form of deposits on the scaffold surface, "trapped" between the scaffold surface and the cell monolayer surface. So, the SPIONs are in contact with the cell surface whether or not the cells are functionalized with the RGD peptide (as can be seen in the confocal microscopy images). Also, in addition to integrins, cells present other surface proteins and membrane structures such as piezo-type calcium channels, gap junctions, or primary cilia, which play a crucial role in mechanosensing of mechanical stimuli [69, 70]. So when the magnetic field is applied to generate a mechanical stimulus through SPIONs, the cells possess several pathways to detect that mechanical stimulus. Therefore, this fact could explain why no increase in differentiation is observed in the condition of scaffolds with SPIONs functionalized with RGD with respect to the condition of scaffolds with SPIONs without RGD.

Nevertheless, after 7 days of culture, an increase in ALP expression was observed in the DMSA 25L condition compared to the RGD 25L condition in both cases applying and not applying magnetic field. This fact was also observed after 14 days of culture in the results of ALP expression without magnetic field and mineralisation after 14 days of culture.

## Discussion

In this work, 3D biofunctional magnetic scaffolds have been successfully fabricated combining FDM printing of a polymeric filament blend of three polymers (PLLA/PCL/PHBV, 90/5/5 wt%) and thermal DOD inkjet printing of SPIONs suspensions on the surface of the previously printed scaffolds. For this purpose, suspensions of SPIONs, prepared by thermal decomposition, stabilised in aqueous medium by ligand exchange and functionalised with an RGD peptide have been used. The SPIONs were incorporated onto the surface of the scaffolds in the form of spots of approximately 100 μm in diameter, faithfully following a designed 2D tetragonal pattern in the circular surface. The surface of the magnetic scaffolds exhibits an increased roughness on the order of 6 times more than the initial one, due to the deposits of these nanomaterials on the spots. The analysed amounts of Fe in the scaffolds demonstrated the accuracy and versatility of the production technique, as well as the stability of the samples after 7 days under physiological conditions. Furthermore, magnetic characterisation indicated that the superparamagnetic properties of the SPIONs are preserved after incorporation on the surface of the polymer scaffolds.

Biological evaluation revealed high viability, adhesion and proliferation of hBM-MSCs in contact with the different scaffolds, demonstrating their high biocompatibility for the intended biomedical applications. Osteogenic differentiation studies using an external magnetic field of 250 mT and 1 Hz frequency for 1 h per day showed a significant increase in ALP gene expression, ALP enzyme activity and mineralisation process in hBM-MSCs. Furthermore, it can be independently distinguished an increase attributed to the presence of SPIONs, an increase related to the application of the magnetic field, and the highest increase when SPIONs are combined with the external magnetic field. Finally, scaffolds with SPIONs functionalised with an RGD peptide targeted to cellular integrins do not enhance the osteogenic effect compared to scaffolds with unfunctionalised SPIONs as it would have been expected. This fact is probably due to the immobilisation of the SPIONs on the scaffold surface so they cannot be targeted to integrin receptors to be internalised via this pathway. Therefore, SPIONs are exposed regardless the RGD motif to other proteins and membrane structures of the cells which also play a crucial role in mechanosensing of mechanical stimuli.

These results together show that the developed 3D magnetic scaffold could be a very promising tool for advanced and personalised bone regeneration treatments. In this sense, the methodology used in this manuscript offers an accurate and precise deposition of magnetic particles over the scaffold surface. Such advantages are important in the context of magnetic scaffolds for bone tissue engineering for several reasons: (i) cells are exposed to a precise and non-cytotoxic amount of SPIONs; (ii) magnetic nanoparticles are homogenously distributed over the scaffold surface, thus exposing cells to the same amount; (iii) SPIONs are deposited following a desired pattern to direct the stimulation to a specific place, and thus regulate a desired biological response, which is an important feature in the context of personalised treatment.

## Supplementary Information

Below is the link to the electronic supplementary material.

Supplementary file1 (DOCX 12797 kb)

## Data Availability

The datasets during and/or analyzed during the current study available from the corresponding author on reasonable request.
