# Supplementary material for: Fabrication of 3D Biofunctional Magnetic Scaffolds by Combining Fused Deposition Modelling and Inkjet Printing of Superparamagnetic Iron Oxide Nanoparticles
Source: Tissue Eng Regen Med. 2025 Mar 18;22(5):627–46. doi: 10.1007/s13770-025-00711-2 (PMC12209090; doi:10.1007/s13770-025-00711-2)
Supplement: Supplementary file 1 — Supplementary file1 (DOCX 12797 kb) [file 13770_2025_711_MOESM1_ESM.docx]

**Supplementary Material**

**Fabrication of 3D biofunctional magnetic scaffolds by combining Fused Deposition Modelling and Inkjet Printing of Superparamagnetic Iron Oxide Nanoparticles**

Manuel Estévez^1,#^, Elisa Batoni^2,3,#^, Mónica Cicuéndez^4^, Amedeo Franco Bonatti^2^, Tamara Fernández-Marcelo^5^, Carmelo De Maria^2,3^*, Blanca González^1,6^, Isabel Izquierdo-Barba^1,6,^*, Giovanni Vozzi^2,3^

*^1^ Departamento de Química en Ciencias Farmacéuticas, Facultad de Farmacia, Universidad Complutense de Madrid, Instituto de Investigación Sanitaria, Hospital 12 de Octubre i+12, Plaza Ramón y Cajal s/n, 28040 Madrid, Spain.*

*^2^ Research Centre “E. Piaggio”, University of Pisa, Pisa, Italy.*

*^3^ Department of Information Engineering, University of Pisa, Pisa, Italy.*

*^4^ Departamento de Química en Ciencias Farmacéuticas, Facultad de Farmacia, Universidad Complutense de Madrid, Instituto de Investigación Sanitaria del Hospital Clínico San Carlos (IdISSC), 28040 Madrid, Spain.*

*^5^ Departamento de Bioquímica y Biología Molecular, Facultad de Farmacia, Universidad Complutense de Madrid, Plaza Ramón y Cajal, s/n, 28040 Madrid, Spain.*

*^6^ Centro de Investigación Biomédica en Red de Bioingeniería, Biomateriales y Nanomedicina (CIBER-BBN), Spain.*

# Both authors contributed equally

* Corresponding authors: Isabel Izquierdo-Barba; Carmelo De Maria

***Equipment***

The iron content of the different SPIONs samples was determined by inductively coupled plasma atomic emission spectroscopy (ICP-AES) in a Perkin Elmer Optima 2100 DV ICP apparatus. Prior to the analysis, samples were digested at 90 °C in HNO_3_/HCl (1:3) and diluted with Milli-Q water.

The crystalline structure of the SPIONs was analyzed by X-ray diffraction (XRD) using a Philips X’Pert diffractometer with Bragg-Brentano geometry operating with CuKα radiation (λ = 1.5406 Å), (Philips Electronics NV, Eindhoven, Netherlands). XRD patterns were collected within 10 and 80 2θ degrees, with a step size of 0.04° and a contact time of 5 s per step.

Particle size and shape were determined by transmission electron microscopy (TEM) using JEOL JEM 1400 instrument operated at 120 kV (JEOL Ltd., Tokyo, Japan). Sample preparation was performed by placing a drop of diluted suspension onto a carbon-coated copper grid.

Fourier transformed infrared (FTIR) spectra were collected in a Thermo Nicolet Nexus spectrometer equipped with a Golden gate attenuated total reflectance (ATR) device (Thermo Fisher Scientific, Waltham, MA, USA).

Chemical microanalysis was performed using Perkin Elmer 2400 CHN and LECO CHNS-932 thermoanalyzers.

A magnetometer with a SQUID type sensor (Superconducting Quantum Interference Device, MPMS-XL type SQUID) was used to measure the magnetic properties of the different materials. For the measurement of liquid samples, 100 µL of sample were placed on a piece of cotton wool, allowed to dry and pressed into a sample holder. To measure the magnetic properties of the scaffolds, the samples were accurately weighed and fitted in the sample holder. The hysteresis loops were measured at 24.8 °C up to 5 T, and the magnetic saturation was obtained by extrapolating to 1/H = 0 the high field of the magnetization curve.

Hydrodynamic size was measured by dynamic light scattering (DLS) from a diluted suspension of the sample in water at pH 7. Electrophoretic mobility measurements for the nanoparticles suspended in water were used to calculate the zeta-potential (ζ) value of the SPIONs. The measurements were performed in a Zetasizer Nano ZS (Malvern Instruments Ltd., United Kingdom) equipped with a 633 nm “red” laser. Samples were measured in quintuplicate (n = 5).

Scanning electron microscopy (SEM) was used to explore the morphological characteristics and surface appearance of the scaffolds, as well as to measure the size of the SPION deposits in a JSM 6335F microscope. For the analysis, the scaffolds were mounted on SEM stubs and coated with carbon under vacuum by sputtering. Images were acquired with an accelerating voltage of 15 kV and different magnifications. In addition, EDS analysis was performed in an Oxford Instruments X-Max 80 mm^2^ model with a resolution of 127 eV at 5.9 KeV.

Atomic Force Microscopy (AFM) was used to explore the nanoscale of the surface of the different scaffolds. A multimode AFM NanoScope III A (Bruker) was used and image processing and calculation of sample roughness (Ra) values were performed with a NanoScope Analysis 2.0.

To evaluate the initial Fe content of the scaffolds, the samples were first digested in HNO_3_/HCl (1:3) and then diluted with Milli-Q water. The Fe concentration was then quantified by ICP-AES. The stability of the samples was studied in 1x PBS (pH 7.4) at 37 °C for 7 days, after which the scaffolds were recovered and the Fe content was measured as above described.

***Reagents***

Sodium oleate 82%, FeCl_3_·6H_2_O 97%, oleic acid 90%, octadecene 90%, dimercaptosuccinic acid 98% (DMSA), dimethyl sulfoxide ≥ 99.9% (DMSO), 12 kDa cellulose membrane, Arg-Gly-Asp (RGD) peptide ≥ 97%, N-(3-dimethylaminopropyl)-N’-ethylcarbodiimide hydrochloride (EDC·HCl) ≥ 98% and *N*-hydroxysulfosuccinimide sodium salt ≥ 98% were purchased from Sigma-Aldrich (Madrid, Spain). Absolute ethanol 99.5%, hexane 95% and toluene 99.5% were purchased from PanReac (Barcelona, Spain). All other chemicals (NaOH, HNO_3_ 65%, HCl 37%, NaCl, MgCl_2_, etc.) were of the highest quality commercially available and used as received. Milli-Q water (resistivity 18.2 MΩ·cm at 25 °C) was used in all experiments.

***Synthetic procedures***

*Synthesis of Iron(III) Oleate.* A total of 45 g of sodium oleate (82%) and 10.8 g of FeCl_3_·6H_2_O (97%) were added to a mixture of 60 mL of distilled water, 80 mL of absolute ethanol and 140 mL of hexane. The mixture was heated to 70 °C and vigorously stirred for 4 h. After cooling down to room temperature, the orange-brown organic phase was washed with ethanol/water mixtures: 1 × (15 mL:35 mL) plus 2 × (25 mL:25 mL). Hexane and traces of water and ethanol were removed in a rotary evaporator resulting in an iron (III) oleate as a dense product that was stored in an oven at 50 °C.

*Synthesis of Iron Oxide Nanoparticles.* In the absence of oxygen, 4.5 g of iron (III) oleate were mixed with 0.71 g of oleic acid (90%) in 50 mL of octadecene (90%) in a 250 mL three-neck round bottom flask equipped with a reflux condenser, mechanical stirring (stirrer shaft), nitrogen flow and thermometer. The mixture was stirred at 340 rpm and heated to boiling temperature. When the temperature reached 50 °C, the stirring was stopped and the nitrogen flow was shut off at 100 °C. Once reflux was reached (315 °C), the reaction was kept at this temperature for 1 h and then allowed to cool to room temperature. The resulting product was washed with ethanol several times until a clean supernatant was obtained (*ca.* 15 × 30 mL using centrifugation cycles at 7500 rpm for 15 min). The material was dried under an air stream and suspended in toluene.

*Transfer to Aqueous Medium by Ligand Exchange.* A suspension of 50 mg of Fe_3_O_4_ nanoparticles in 20 mL toluene was added to a solution of 200 mg of DMSA (98%) in 5 mL of DMSO. The obtained suspension was placed in several glass vials that were shaken on a carousel (rotary shaker) at a constant speed for 3 days. DMSA-coated nanoparticles precipitated adhering to the wall of the glass vial, and the supernatant was discarded. The nanoparticles were then recovered and washed 4 times with ethanol by centrifugation at 7500 rpm for 20 min. The black solid was dried under an air stream and suspended in 5 mL of distilled water. The pH of the suspension was adjusted to 10 with 2 M NaOH and dialyzed against distilled water for 72 h using a 12 kDa cellulose membrane. Finally, the pH of the colloidal suspension was adjusted to 7.

*Conjugation of the RGD peptide to the nanoparticles.* RGD peptide was covalently attached to the carboxyl groups present in the DMSA coating of the SPIONs through EDC/sulfoNHS chemistry following the procedure reported by Herranz [^[[1]](#endnote-1)^]. In brief, 25 mg of EDC·HCl and 30 mg of sulfo NHS were added to 10 mL of aqueous suspension of nanoparticles at a concentration of [Fe = 1 mg/mL]. The mixture was stirred at room temperature for 2 h and then purified by centrifugation at 12000 rpm and 4 °C for 20 min. The nanoparticle pellet was diluted in Milli-Q water to 15 mL, 2 mg of peptide was added, and the mixture was stirred at room temperature overnight. The sample was then purified by centrifugation (twice) and suspended in Milli-Q water.

*Novel printing platform.* The 3D scaffolds without/with SPIONs were fabricated with a novel printing platform featuring a module for the Fused Deposition Modelling (FDM) printing, and a thermal Drop-On-Demand (DoD) inkjet module for the deposition of low viscous solutions (Figure S1). The platform is controlled through LinuxCNC, open-source software for controlling CNC machines (*e.g.*, lathes, 3D printers), which enables the real-time control of the positioning system and the easy integration of new modules with the previously existing ones [^[[2]](#endnote-2)^,^[[3]](#endnote-3)^].


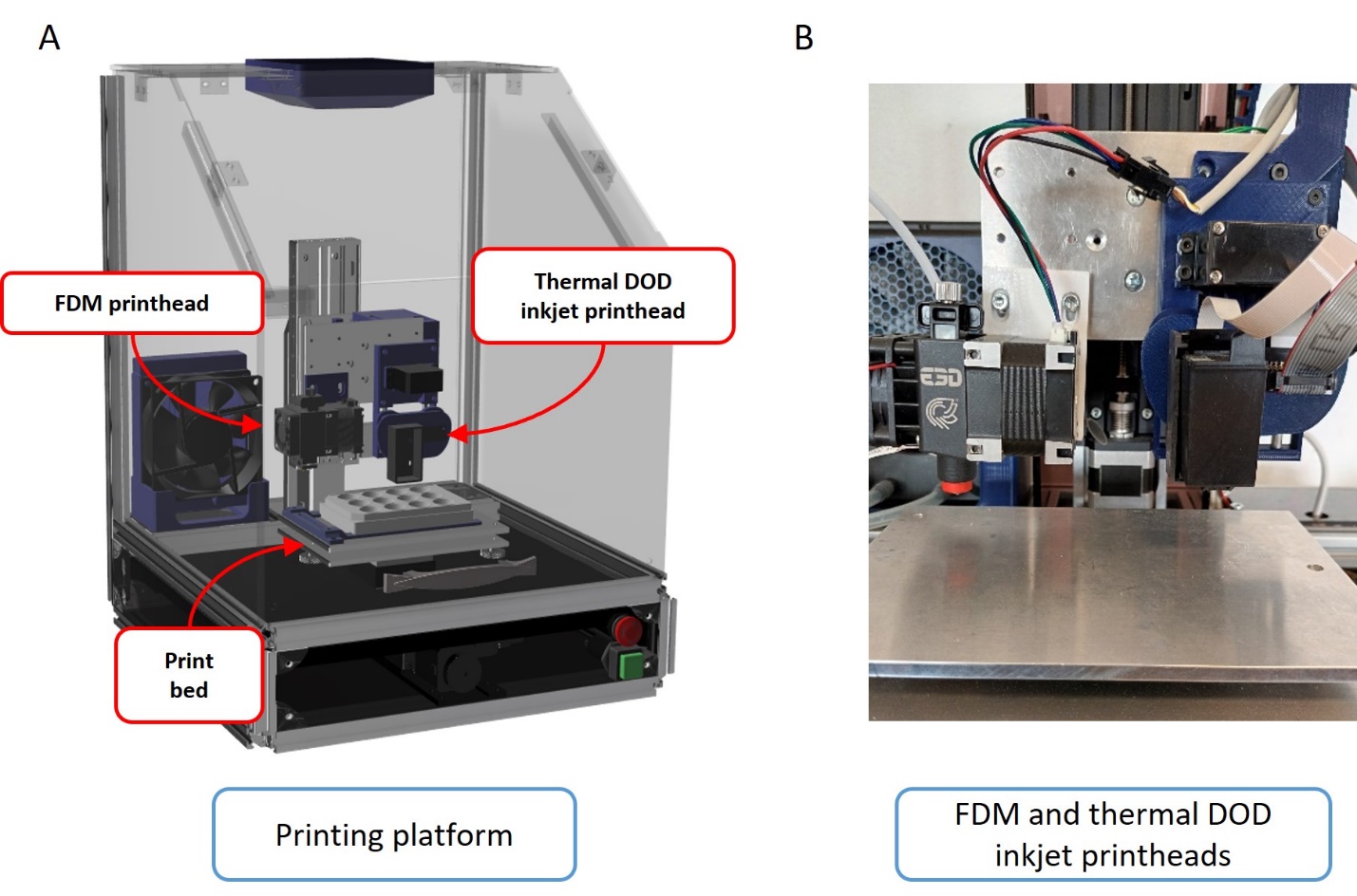


***Figure S1.*** *Novel printing platform: A) a rendering of the developed platform, and B) a picture of the printing tools, namely, the FDM and inkjet printheads.*

Video S1: <https://drive.google.com/file/d/1rnmV7fvavSCo1WmIFtT2-8hgFvq9jGS8/view?usp=drive_link>

Video S2: <https://drive.google.com/file/d/1-f1T2UImuZMWxi0zns1WCt2xk6DaJI_6/view?usp=drive_link>

*Preparation of SPIONs suspensions.* SPIONs were prepared by exploiting the thermal decomposition of iron (III) oleate as an iron-containing precursor [^[[4]](#endnote-4)^]. After synthesis, structural characterization of the oleic acid-stabilized nanoparticles (SPIONs-OA) was performed. The XRD pattern (Figure S2A) showed multiple peaks consistent with the inverse spinel structure of magnetite [^[[5]](#endnote-5)^], TEM analysis (Figure S2B) confirmed a uniform nanoparticle size, with dimensions of about 11 nm. The nanoparticles were further processed by exploiting a ligand exchange of the oleic acid surface layer with DMSA to obtain a stable suspension of nanoparticles in water (SPIONs-DMSA) [4]. The FTIR spectrum (Figure S4A) confirmed the success of the ligand exchange. Next, SPIONs-DMSA nanoparticles were covalently functionalized with an Arg-Gly-Asp (RGD) peptide via amide bonds (Figure S3). For this purpose, the carboxylic acid groups present in the DMSA coating of the SPIONs were activated through EDC/sulfoNHS chemistry and reacted with the primary amine of the RGD sequence. The functionalization was verified by FTIR, DLS (Figure S4A-B) and chemical microanalysis (Table S1) [^[[6]](#endnote-6)^]. The magnetization curves of the synthesized SPIONs showed absence of hysteresis loop, therefore confirming their superparamagnetic behavior (Figures S2C and S4C).


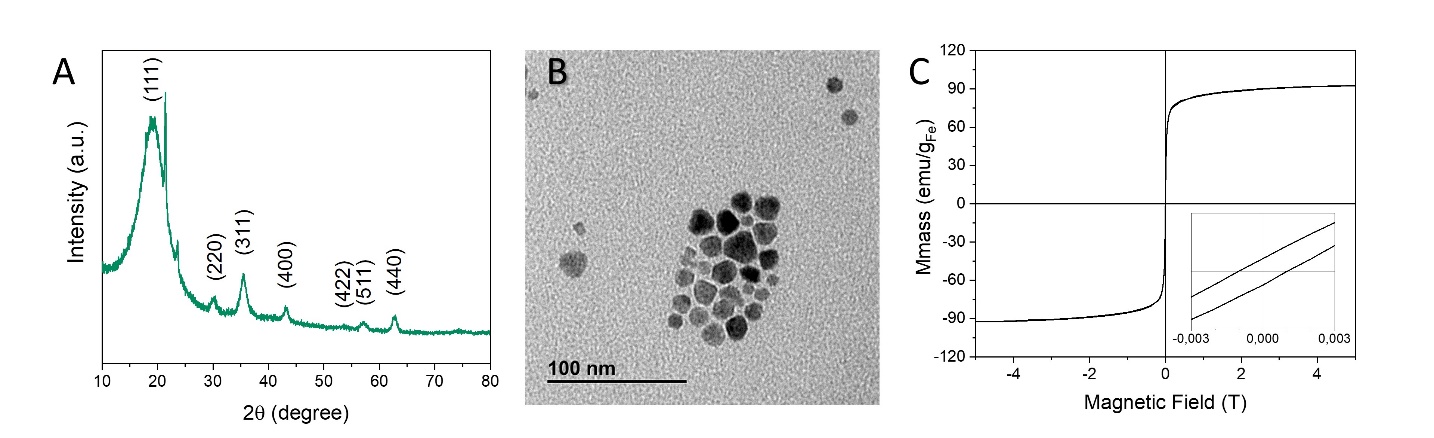


***Figure S2.*** *A) Powder X-ray diffraction pattern, B) TEM image and C) magnetization curve normalized to the grams of iron of as-synthetized SPIONs-OA. Inset in C) magnification of the magnetization curve at low fields.*

***Figure S3.*** *Scheme of synthesis of the functionalization of SPIONs with RGD peptide. Carboxylic acid activation of SPIONs-DMSA via carbodiimide chemistry and subsequent anchoring of RGD peptide via amide bonds.*

***Table S1.*** *Elemental composition from chemical analyses (atomic percentages) of DMSA-coated and RGD-functionalized SPIONs.*

| **Sample** | **% C** | **% H** | **% N** | **% S** |
| --- | --- | --- | --- | --- |
| **SPIONs-DMSA** | 3.93 | 0.63 | 0.22 | 3.45 |
| **SPIONs-DMSA-RGD** | 4.53 | 1.30 | 0.78 | 0.60 |


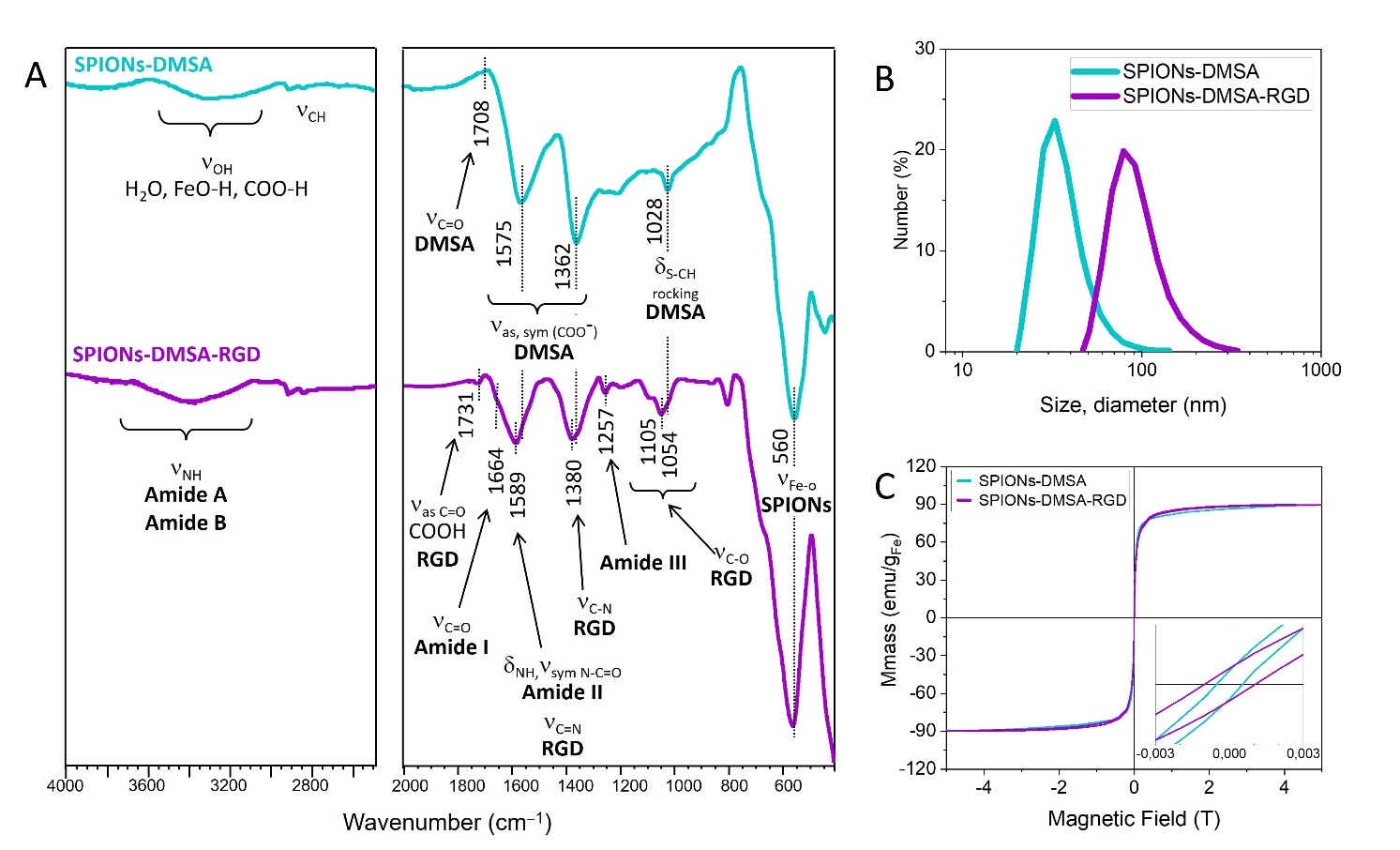


***Figure S4.*** *A) FTIR spectra of DMSA-coated (SPIONs-DMSA) and functionalized with the RGD peptide (SPIONs-DMSA-RGD) nanoparticles. B) Size distributions obtained by dynamic light scattering in water media of SPIONs-DMSA and SPIONs-DMSA-RGD nanoparticles. C) Magnetization curves of SPIONs-DMSA and SPIONs-DMSA-RGD samples normalized to the mass of iron. The magnification of the magnetization curves near the origin are shown as inset.*

***Figure S5.*** *Magnetization curves of scaffold samples normalized to the mass of iron. The magnification of the magnetization curves near the origin are shown as inset.*

***Figure S6.*** *Top: SEM images and EDS mapping, performed in field emission JSM 6335F microscope, corresponding to the surface of different scaffolds DMSA and RGD after 25- and 50-layer deposition, respectively. The iron distribution onto the surfaces is represented in red. Bottom: high resolution SEM micrographs obtained with a JSM7600 microscope showing the SPIONs deposits of DMSA 25L, RGD 25L and DMSA 50L scaffolds as representative sample, respectively.*


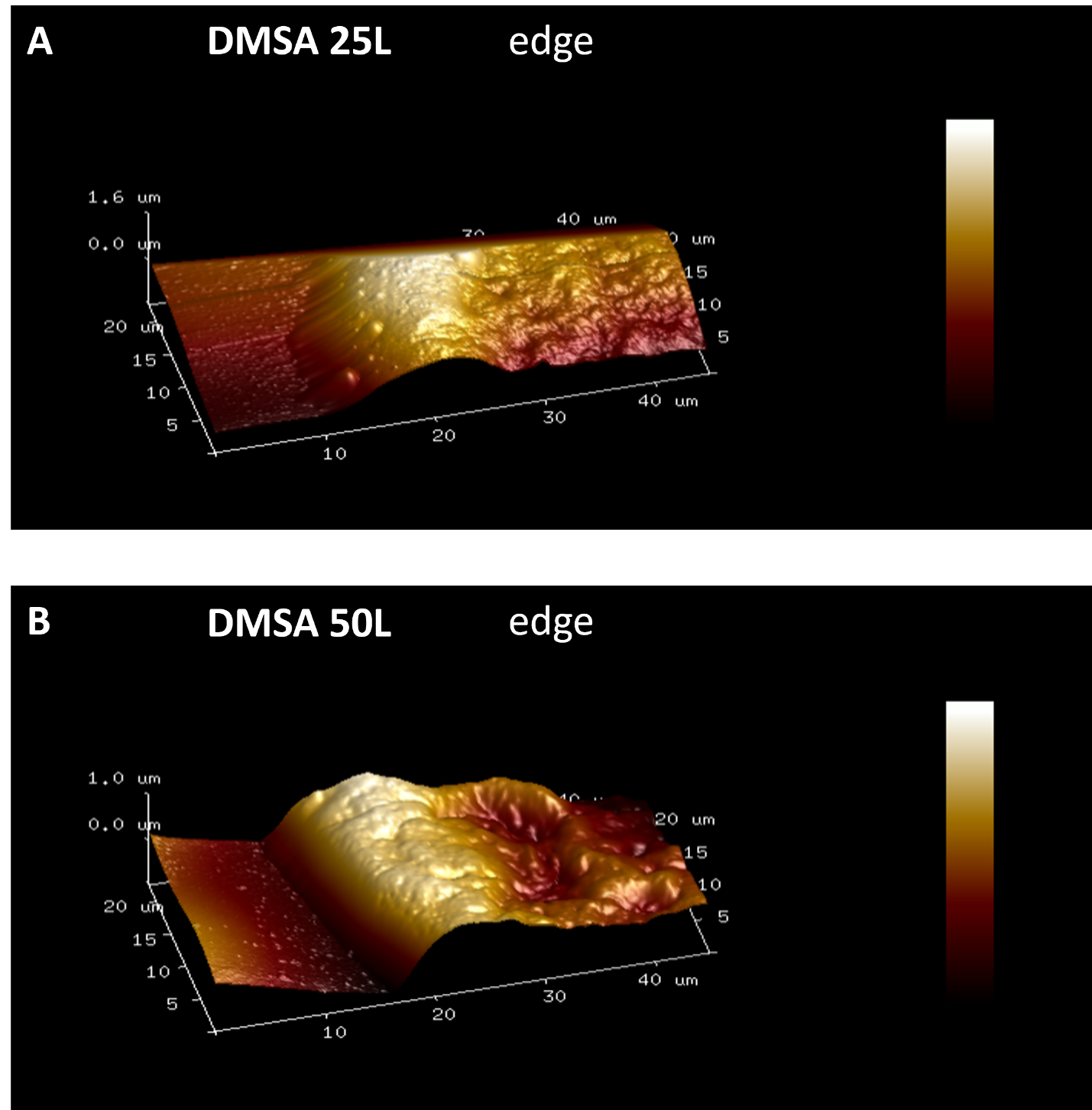


***Figure S7.*** *3D AFM images of the DMSA 25L and 50L scaffolds measured in the area between the drop and the surface to determine the height difference between the two compartments. An area of 45 μm x 20 μm was scanned. The results are in good agreement between both types of nanoparticles (DMSA and RGD SPIONs), so the results for DMSA scaffolds are shown to be representative. Both types of samples present a step of approximately 1.3 μm that decreases for the 25L case returning almost to its baseline and is maintained for the 50L case, with a thicker droplet appearing due to the higher amount of nanoparticles. These results agree with the ICP analysis of the samples for the different depositions.*

***Figure S8.*** *Representative SEM images of hBM-MSCs cultured on SC scaffolds (A and F), DMSA 25L (B and G), RGD 25L (C and H), DMSA 50L (D and I) and RGD 50L (E and J) after 14 days in culture. The magnifications used were ×500 (A to E) and x1000 (F to J).*

***Figure S9.*** *SEM images of hBM-MSCs cultured on the different scaffolds: SC (A), DMSA 25L (B), RGD 25L (C), DMSA 50L (D) and RGD 50L (E) after 14 days in culture. White arrows indicate SPIONs deposits found under the cell monolayer. The magnifications used were ×30.*

***Figure S10.*** *Evaluation of Runx2 gene expression in hBM-MSCs cultured on the surface of SC, DMSA 25L and RGD 25L scaffolds by applying (+ MF) and not applying (- MF) an external magnetic field of 1 Hz frequency for 1 h per day. Expression was measured after 7 days. Results were statistically analyzed by one-way ANOVA and Tukey's test.*

**REFERENCES**

1. [] Herranz F, Morales MP, Roca AG, Vilar R, Ruiz‐Cabello J. A new method for the aqueous functionalization of superparamagnetic Fe2O3 nanoparticles. Contrast Media & Molecular Imaging 2008;3(6):215-222. [↑](#endnote-ref-1)
2. [] Staroveški T, Brezak D, Udiljak T. Linuxcnc--the enhanced machine controller: application and an overview. Tehnicki vjesnik/Technical Gazette 2013;20(6):1103-1110. [↑](#endnote-ref-2)
3. [] Fortunato GM, Batoni E, Bonatti AF, et al. Surface reconstruction and tissue recognition for robotic-based in situ bioprinting. Bioprinting. 2022;26:e00195. [↑](#endnote-ref-3)
4. [] Salas G, Casado C, Teran FJ, Miranda R, Serna CJ, Morales MP. Controlled synthesis of uniform magnetite nanocrystals with high-quality properties for biomedical applications. Journal of Materials Chemistry 2012;22(39):21065-21075. [↑](#endnote-ref-4)
5. [] Wu W, Wu Z, Yu T, et al. Recent progress on magnetic iron oxide nanoparticles: synthesis, surface functional strategies and biomedical applications. Sci Technol Adv Mater. 2015;16:23501. [↑](#endnote-ref-5)
6. [] Estévez M, Cicuéndez M, Colilla M, et al. Magnetic colloidal nanoformulations to remotely trigger mechanotransduction for osteogenic differentiation. J Colloid Interface Sci. 2024;664:454–68. [↑](#endnote-ref-6)
